# Insects to feed insects - feeding *Aedes* mosquitoes with flies for laboratory rearing

**DOI:** 10.1038/s41598-019-47817-x

**Published:** 2019-08-06

**Authors:** Nanwintoum Séverin Bimbilé Somda, Hamidou Maïga, Wadaka Mamai, Hanano Yamada, Adel Ali, Anna Konczal, Olivier Gnankiné, Abdoulaye Diabaté, Antoine Sanon, Kounbobr Roch Dabiré, Jérémie R. L. Gilles, Jérémy Bouyer

**Affiliations:** 1 Insect Pest Control Laboratory, Joint FAO/IAEA Division of Nuclear Techniques in Food and Agriculture, International Atomic Energy Agency, A-1400 Vienna, Austria; 2 Institut de Recherche en Sciences de la Santé/Direction Régionale de l’Ouest (IRSS/DRO), 01 BP 545 Bobo-Dioulasso, Burkina Faso; 3 Laboratoire d’Entomologie Fondamentale et Appliquée, Université Joseph Ki-Zerbo, 03 BP 7021, Ouagadougou, Burkina Faso; 4 Institut de Recherche Agricole pour le Développement (IRAD), BP 2123 Yaoundé-Messa, Cameroon

**Keywords:** Genetic techniques, Diseases

## Abstract

The black soldier fly, yellow mealworm and house fly are known for their wide distribution, ease of breeding, and environmental and nutritional attributes. Diets based on these fly proteins for the rearing of mosquito larvae are more accessible and affordable when compared to the reference IAEA diet which consists largely of costly livestock products such as bovine liver powder. Following a step-by-step assessment, we developed diet mixtures based on insect meal for the optimal mass production of *Aedes albopictus* and *Ae. aegypti*. Based on the assessed parameters including mosquito egg hatch, body size, flight ability, longevity and diet cost reduction, two mixtures are recommended: 1/2 tuna meal (TM) + 7/20 black soldier fly (BSF) + 3/20 brewer’s yeast and 1/2 TM + 1/2 BSF. These findings, which could be adapted to other mosquito species, provide alternative protein sources for mass rearing insects for genetic control strategies.

## Introduction

Human health is facing several burdens, including vector-borne diseases such as dengue, chikungunya, yellow fever and zika^1,2^. Between 67–136 million dengue virus infections are estimated to occur each year^3^. Early in 2016, Zika virus disease was declared a public health emergency of international concern with 500,000 suspected cases in America^4,5^. *Aedes aegypti* Linnaeus, 1762 (Diptera: Culicidae), and *Ae. albopictus* Skuse, 1894 (Diptera: Culicidae) mosquitoes are main vectors and efficiently transmit these diseases. *Aedes aegypti* is known to be a major vector with a wide distribution throughout the world while *Ae. albopictus* has potential to invade new regions^6–8^ including urban areas^9^. Conventional methods to treat or to prevent these diseases and their vectors are not sufficient to prevent their spread. Particularly, the use of insecticides as the main vector control method is promoting the emergence of insecticide resistance and alterations in behavior in vector populations. Furthermore, insecticides are a major human, animal and environmental health concerns^10,11^. Alternative and safer methods are therefore needed. Thus the interest for biological approaches such as the sterile insect technique (SIT) is growing^12^.

The SIT is a species-specific technique based on the release of irradiated sterile males which transfer the sterility to wild females via mating, inducing a progressive decline of the target population. This technique is environment-friendly and can be combined with other methods as part of an area-wide integrated pest management approach^13–15^. It requires the production of a large quantity of males to be released. Based on mating, its success is conditioned by the quality of reared males. Mating capacity and fecundity is positively correlated to adult body size in *Ae. aegypti* and *Ae. albopictus*^16,17^ while the characteristics of adult mosquitoes are determined by the quality of larval diet^18,19^. Hence, the quality of larval diet is a key driver to provide sufficient and efficient males. Ecological investigations revealed that natural food resources of *Aedes* species include microorganisms, detritus and invertebrates^20^. For laboratory rearing, artificial diets have been developed by mixing products from livestock, cereals, yeast and algae^21–23^. The diet used for *Aedes* larval rearing at the Insect Pest Control Laboratory (IPLC) of the Joint FAO/IAEA is a mixture of tuna meal (TM), bovine liver powder (BLP) and brewer’s yeast (BY)^24^. The current larval diets are facing food security concerns, including availability, access, utilization and stability, especially in developing countries. To create affordable and sustainable mosquito control techniques based on mass production, new sources of accessible ingredients are urgently required. Edible insects are among the potential alternatives to explore. Here we show that they can indeed constitute an appropriate and cost-effective ingredient to rear mosquito larvae.

Insects have been a reliable food source in many countries for centuries. While acceptance of many edible insects as food for humans continues to be discussed due to cultural and religious commitments^25–27^, their opportunity to be used to feed animals has become widely accepted. Veldkamp *et al*.^28^ reported that insects can be reared at large scale and used as an alternate sustainable rich protein in the diet for pets and livestock. *Hermetia illucens* Linnaeus, 1758, (Diptera: Stratiomyidae), known as the black soldier fly (BSF), *Tenebrio molitor* Linnaeus, 1758 (Coloeoptera: Tenebrionidae), known as the mealworm beetle, and *Musca domestica* Linnaeus, 1758 (Diptera: Muscidae), known as the house fly (HF), count among those insects that have received attention, due to their distribution, ease of breeding, and environmental and nutritional attributes. BSF can be reared on organic waste. The adults do not feed and rely on the fat stored from the larval stages^29^. The complete life cycle takes 1–2 months^30^. The larvae can be easily dried for longer storage^28^. BSF larvae contain 40–44% crude protein and a variable fat amount (15–49%) depending on the type of diet^31,32^. Mealworm beetle’s life cycle varies in length and can last 280 to 630 days. Its larva, also called yellow mealworm (YM), is omnivorous, can eat all kinds of plant materials as well as animal products and can recycle plant waste materials of low quality into a high-quality feed^33^. Dried YM contains 47–60% of crude proteins and 31–43% of fat^34^. HF is the most common fly species. Its larva (maggot) and adult feed on manure and decaying organic wastes and convert these into valuable biomass rich in proteins and fat with a life cycle lasting 6 to 10 days. HF maggot contains between 40 and 60% crude protein and 9 to 26% of fat. BSF, YM, and HF meal are known to be effective components in the diet of ruminants, pigs, poultry and fish species^35^ and are therefore potential diet ingredients for aquaculture including mosquito rearing.

The present study investigated the possibility to use insect meal (IM) in *Ae. aegypti* and *Ae. albopictus* larval diet for optimal cost/yield mass rearing. Ten different IM (Table 1) from BSF, YM, and HF provided by different suppliers were assessed. Initially, each individual insect meal was evaluated on its own for its effect on larval development. Thereafter, the effect of each IM used in place of BLP in the reference IAEA diet was assessed. Furthermore, optimal mixtures of TM, BY and IM were determined. Finally, the four most promising mixtures were selected and assessed on both larva and adult mosquito life history traits including fecundity, egg hatch, survival, body size and male flight ability.

**Table 1:** Description of insect meal.

Insect meal	Insect species	Insect common name	Development instar	Type of dry mass	Supplier
A	*Hermetia illucens*	BSF	Larvae	Non-defatted	InnovaFeed
B	*Hermetia illucens*	BSF	Larvae	Defatted	InnovaFeed
C	*Hermetia illucens*	BSF	Pre-pupae	Non-defatted	InnovaFeed
D	*Hermetia illucens*	BSF	Larvae	Non-defatted	InnovaFeed
E	*Hermetia illucens*	BSF	Larvae	Non-defatted	InnovaFeed
F	*Hermetia illucens*	BSF	Pre-pupae	Non-defatted	InnovaFeed
G	*Hermetia illucens*	BSF	Larvae	Defatted	Protix
H	*Tenebrio molitor*	YM	Larvae	Non-defatted	Protix
I	*Tenebrio molitor*	YM	Larvae	Protein	Protix
J	*Musca domestica*	HF	Larvae	Protein	Protix

Insect meal C and F differ by the type of feed used during their rearing. Insect meal A, D and E differ from the process of grinding. BSF: Black soldier fly; YM: Yellow mealworm; HF: House fly.

## Results

### Effect of pure insect meal diet on *Aedes albopictus* larval development

**Figure 1:**
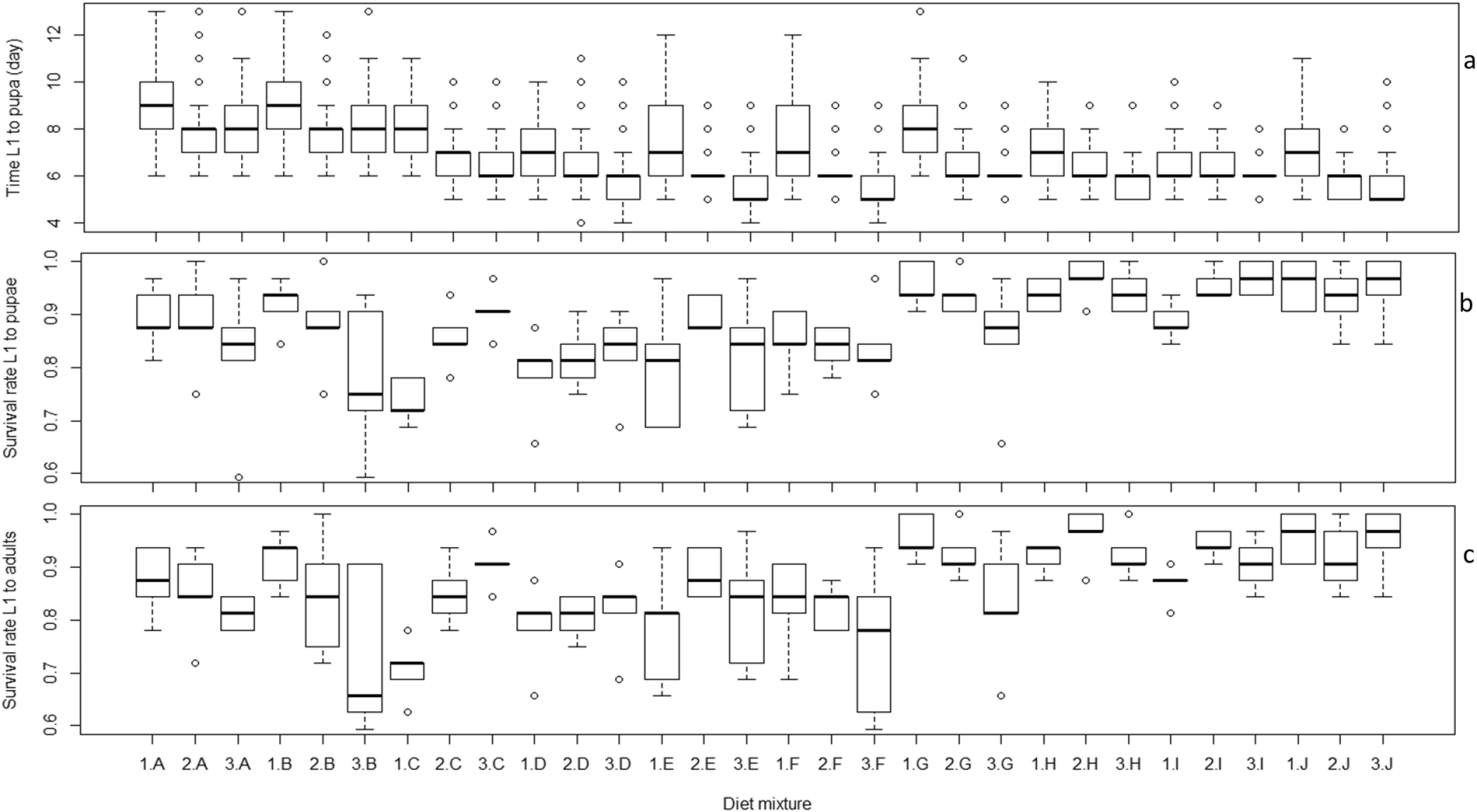
Effect of pure insect meal on *Aedes albopictus* larval development time to pupation (**a**), survival rate to pupae (**b**) and to adults (**c**). On the X-axis, the numbers 1–3 correspond to the diet concentration and the letters A-J correspond to the insect meal used.

All pure IM diet treatments allowed a complete development of *Ae. albopictus* larvae to adult emergence. Overall, larval stages were completed between 5 and 10 days with high larval survival rates from L1 to pupae and to adults (70–98%) (Fig. 1). Compared to IM diet H which seems to result in the highest larval survival rate from L1 to adults (Fig. 1), IM diets G, I and J had similar survival rates (*P* > 0.05) while IM diets A through F significantly reduced the survival rate (*P* < 0.05) (Tables S1 and S2). However, IM diet J resulted in the shortest larval development time (*P* < 0.001) while IM diets E, F, G and I were similar to IM H (*P* > 0.05) followed by IM diets A through D which significantly increased it (*P* < 0.001) (Table S3). Larval survival rates from L1 to pupae and to adults were not affected by the diet concentration. (*P* > 0.05) (Tables S1 and S2). However, a higher diet concentration resulted in shorter larval development times (*P* < 0.05) (Table S3). The interaction between insect meal and diet concentration was also significant across all assessed parameters (Tables S1–S3).

### Effect of insect meal substituting BLP in the reference IAEA diet mixture on *Aedes albopictus* larval development

**Figure 2:**
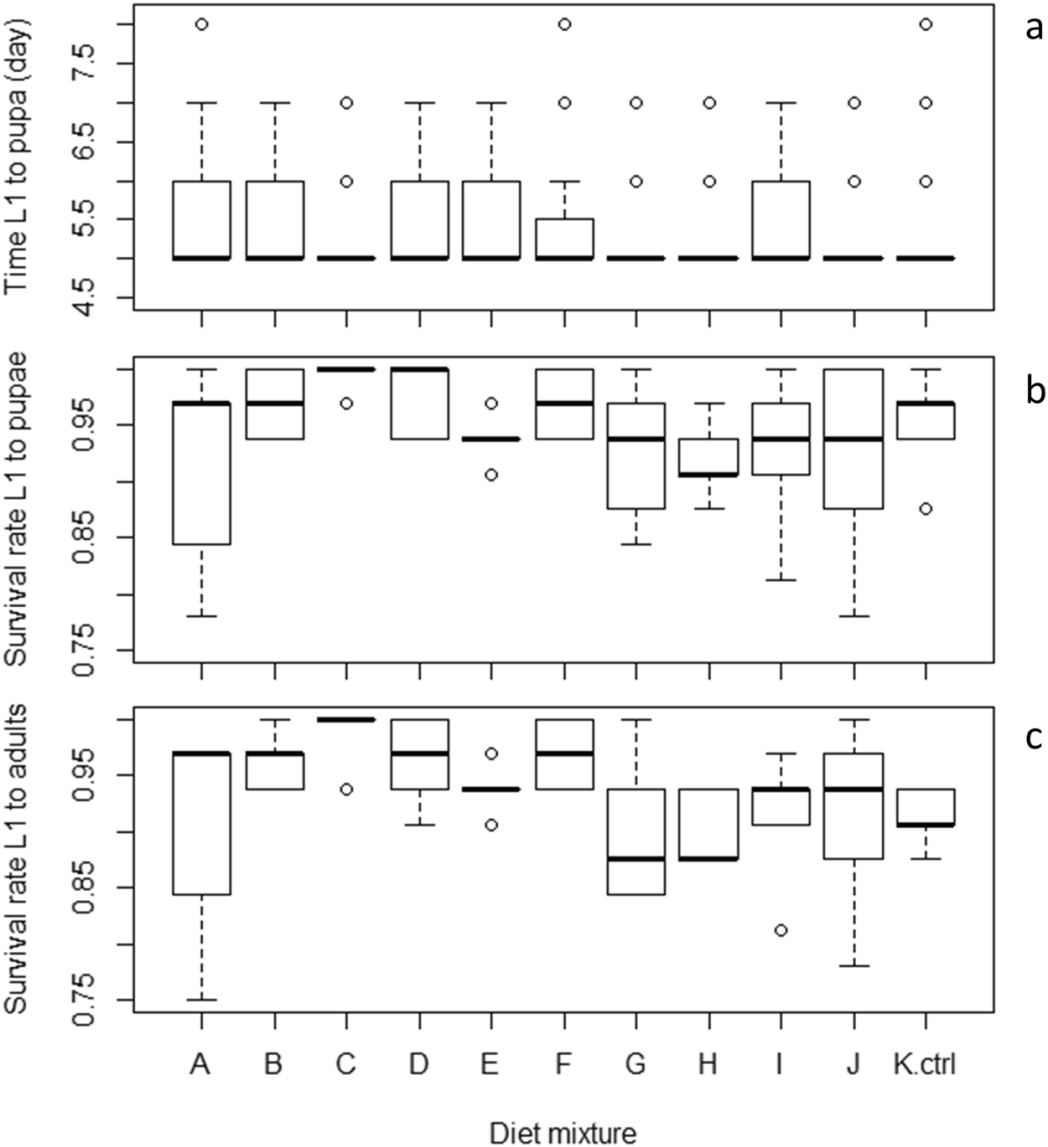
Effect of insect meal as a substitution of BLP on *Aedes albopictus* larval development time to pupation (**a**), survival rate to pupae (**b**) and to adults (**c**). On the X-axis, the letters A-J correspond to the insect meal used as a substitution of BLP in the IAEA reference diet. BLP: Bovine liver powder; IAEA: International Atomic Energy agency.

Following the substitution of BLP by the different IM in the reference IAEA diet, the mixtures were renamed Mix A to Mix J, and Mix K for the control (reference IAEA diet). All treatments allowed *Ae. albopictus* larvae to complete their development from L1 to adult with short median larval development times to pupation (around 5 days) and high median larval survival rates (>90%) (Fig. 2). Compared to the control (K), Mix A, B, D, E and I significantly increased the larval development time (*P* < 0.05), Mix C and J accelerated development (*P* < 0.05), and Mix F, G, H showed no effect (*P* > 0.05) (Table S4). Mix C resulted in a higher larval survival rate to pupae (*P* < 0.05) and Mix C and F induced higher larval survival rates to adults (*P* < 0.05) while the other mixtures were statistically similar to the control (K) (*P* > 0.05) (Table S5).

### Optimal diet mixtures of tuna meal (TM), brewer’s yeast (BY) and insect meal (IM)

The non-defatted pre-pupae of BSF (named IM-C or BSF-C) and the defatted larvae of BSF (named IM-G or BSF-G) were selected for the determination of optimal diet combinations. The insect meal was selected mainly on the basis of the results of experiments 1 and 2 and also because BSF species has a shorter life cycle than YM and is not a pest conversely to HF. In addition, proteins requiring extraction would be more expensive than the other meal. Three-component mixture design experiments were conducted considering TM, BY and BSF-C (design 1) on one hand and TM, BY and BSF-G on the other hand (design 2).

**Figure 3:**
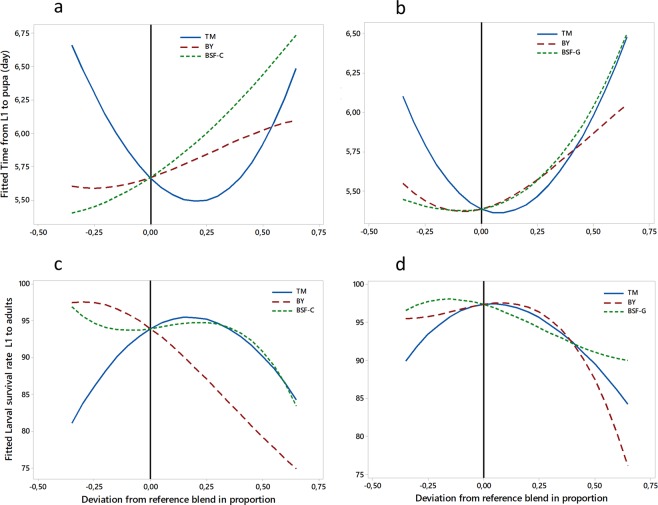
The effect of each component in different mixture designs on *Aedes aegypti* larval development parameters. The top panels present the impact on larval development time from L1 to pupae in the mixtures TM, BY, BSF-C (**a**) and TM, BY, BSF-G (**b**). The bottom panels present the impact on larval survival rate from L1 to adults with mixtures TM, BY, BSF-C (**c**) and TM, BY, BSF-G (**d**), respectively. The reference blend is the mixture composed of equal amounts (1/3) of each of the three ingredients. TM: Tuna meal; BY: Brewer’s yeast; BSF-C: Black soldier fly corresponding to insect meal C; BSF-G: Black soldier fly corresponding to insect meal G.

**Figure 4:**
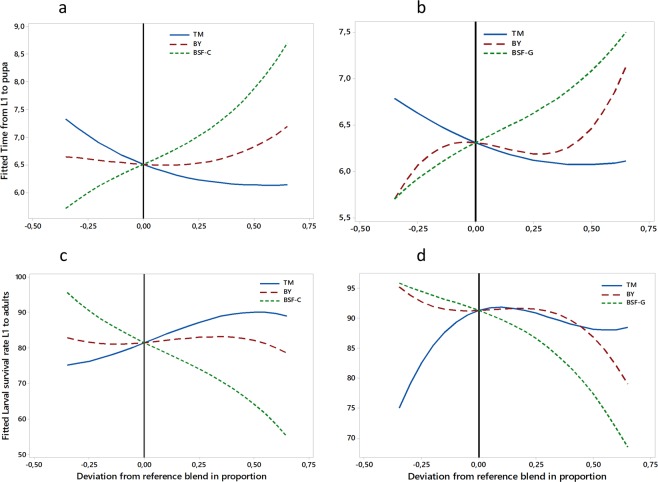
Effect of each component in each mixture design on *Aedes albopictus* larval development parameters. The top panels present the impact on larval development time from L1 to pupae in the mixtures TM, BY, BSF-C (**a**) and TM, BY, BSF-G (**b**). The bottom panels present the impact on larval survival rate from L1 to adults in the mixtures TM, BY, BSF-C (**c**) and TM, BY, BSF-G (**d**). The reference blend is the mixture composed of equal amounts (1/3) of each of the three ingredients. TM: Tuna meal; TM: Tuna meal; BY: Brewer’s yeast; BSF-C: Black soldier fly corresponding to insect meal C; BSF-G: Black soldier fly corresponding to insect meal G.

The trace plots show how each component affects the response relative to the reference blend (equal amount of the three components: 1/3 each) in the mixture designs 1 and 2 on *Ae. aegypti* (Fig. 3) and *Ae. albopictus* (Fig. 4) larval development parameters. Overall, in both mixtures and both species, the greater the proportion of BSF (C or G), the longer the larval development time to pupation and the lower the larval survival rate to adults. The effect of BY followed the same trend. However, an increase or a decrease in TM proportions led to longer larval development time and lower larval survival rate to adults, with its optimal proportion being slightly higher than that in the reference blend (1/3).

Detailed description of regressions for mixtures for both *Aedes* species development parameters versus both mixture designs is presented in Tables S6–S13.

#### The regression for mixtures of *Ae. aegypti* larval survival rate versus TM, BY, BSF-C and versus TM, BY, BSF-G

Whatever the design (Tables S6 and S7), conversely to the three-component blend, all the two-component blends had a positive effect on *Ae. aegypti* larval survival rate. Significant effects (*P* < 0.05) were observed with TM*BY and TM*BSF-C, TM*BSF-G, BY*BSF-G but not with BY*BSF-C. The two-component blend TM*BY had the highest magnitude of the coefficients followed by those of TM*BSF. The association TM*BY had then the best synergy.

#### The regression for mixtures of *Ae. aegypti* larval development time from L1 to pupa versus TM, BY, BSF-C and versus TM, BY, BSF-G

The two-component blends, TM*BY, TM*BSF-C, TM*BSF-G, BY*BSF-G (Tables S8 and S9), were found to act significantly (P < 0.05) to reduce *Ae. aegypti* larval development time to pupation with higher effect of TM*BSF. The impact of the three-component blend was not significant (P > 0.05). TM*BSF had the best impact on larval developmental time followed by TM*BY.

#### The regression for mixtures of *Ae. albopictus* larval survival rate versus TM, BY, BSF-C and versus TM, BY, BSF-G

In both mixture designs, TM had the highest magnitude of the coefficients among the pure mixtures followed by BY. Positive coefficients were observed with all two-component blends indicating that they acted synergistically, conversely to the three-component blends. The interaction TM*BSF had the highest magnitude of the coefficients followed by the TM*BY. Only the effects of TM*BY and TM*BSF-G were significant (*P* < 0.05) among the two-component blends. Based on the magnitude of the coefficients, the association TM*BSF was the best (Tables S10 and S11).

#### The regression for mixtures of *Aedes albopictus* larval development time from L1 to pupa versus TM, BY, BSF-C and versus TM, BY, BSF-G

All two-component blends acted significantly for the reduction of *Ae. albopictus* larval development time from L1 to pupa (*P* < 0.05). The highest impact was observed with TM*BSF followed by TM*BY. Conversely, the three-component blend showed an increase but not significantly (*P* > 0.05) (Tables S12 and S13). The two-component blend TM*BSF was therefore the best. Regardless of the design and the species, the two-component mixtures TM*BY and TM*BSF significantly increased the larval survival rate and reduced the larval development time from L1 to pupa (*P* < 0.05). Since the effects of the three-component mixtures were not significant and likely revealed an antagonistic interaction between ingredients, we focused interpretation of the contour plots only on the two-component mixtures which showed significant and synergic effects.

#### *Aedes aegypti* larval development time from L1 to pupa

**Figure 5:**
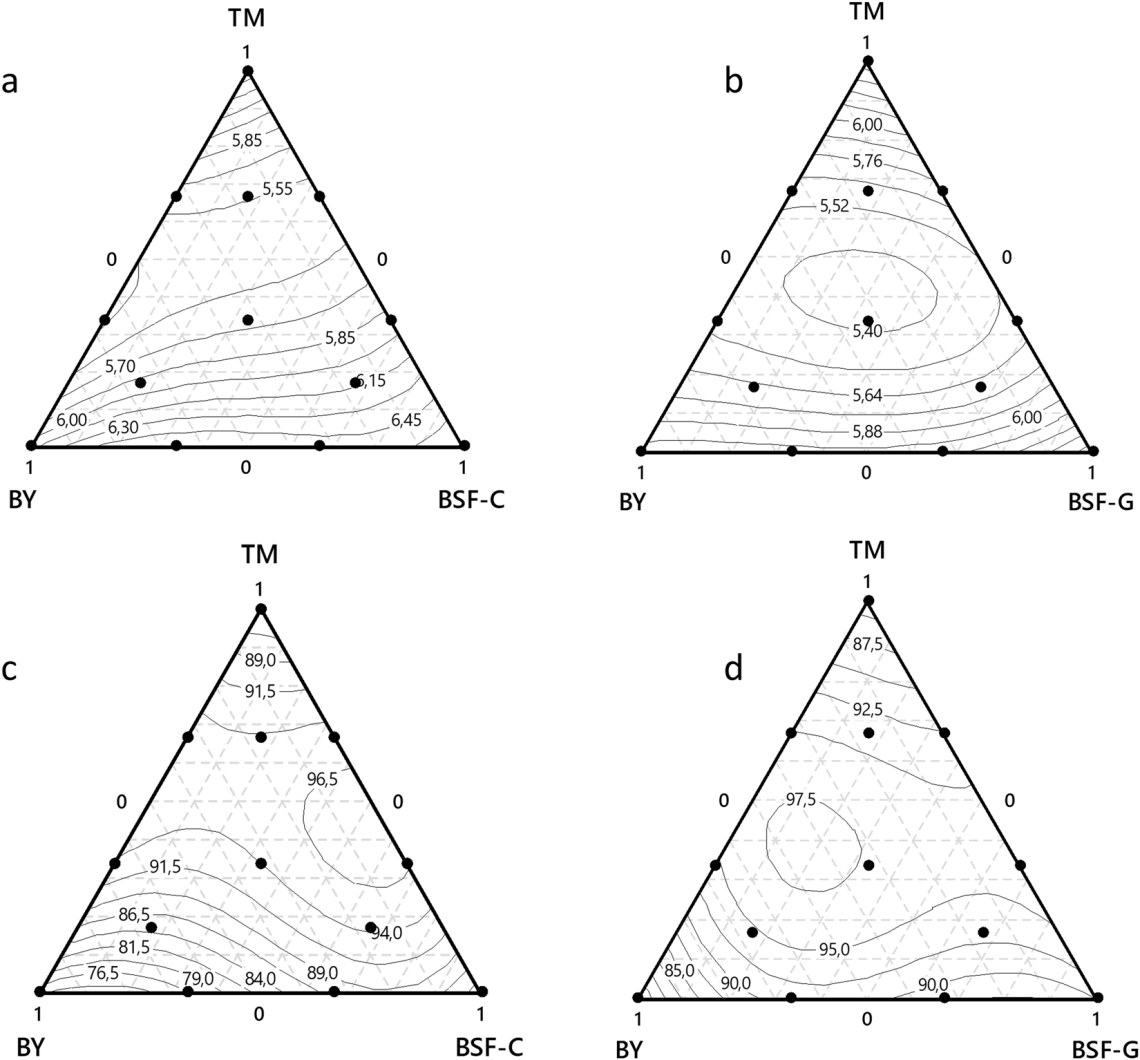
Effects of diet mixtures with different mixture designs on *Aedes aegypti* larval development parameters. The top panels present the impact on larval development time from L1 to pupae in the mixtures TM, BY, BSF-C (**a**) and TM, BY, BSF-G (**b**). The bottom panels present the impact on larval survival rate from L1 to adults with the mixtures TM, BY, BSF-C (**c**) and TM, BY, BSF-G (**d**). In these plots, the response surface is viewed as a two-dimensional plane where all blends that have the same response are connected to produce contour lines of constant responses. TM: Tuna meal; BY: Brewer’s yeast; BSF-C: Black soldier fly corresponding to insect meal C; BSF-G: Black soldier fly corresponding to insect meal G.

All mixtures composed of TM + BSF and TM + BY resulted in larval development times ranging between 5.40 and 6 days. The two-component blend BSF + BY resulted in the longest larval development times (>6 days) (Fig. 5a,b).

#### *Aedes aegypti* larval survival rate from L1 to adults

In the design with TM, BY and BSF-C, the two-component blends, 1/2TM + 1/2 BSF-C and 1/3TM + 2/3 BSF-C induced the highest larval survival rate to adults (>96.5%) followed by 2/3TM + 1/3BSF-C, 1/2TM + 1/2 BY, 2/3 TM + 1/3 BY and 1/3 TM + 2/3 BY (between 94 and 96.5%) (Fig. 5c).

In the design with TM, BY and BSF-G, the two-component blends,1/2TM + 1/2 BY and 2/3 TM + 1/3 BY, 1/3TM + 2/3BY, 1/3TM + 2/3BSF-G and 1/2TM + 1/2BSF-G had the highest larval survival rate (between 95–97.5%) (Fig. 5d).

#### *Aedes albopictus* larval development time from L1 to pupae

**Figure 6:**
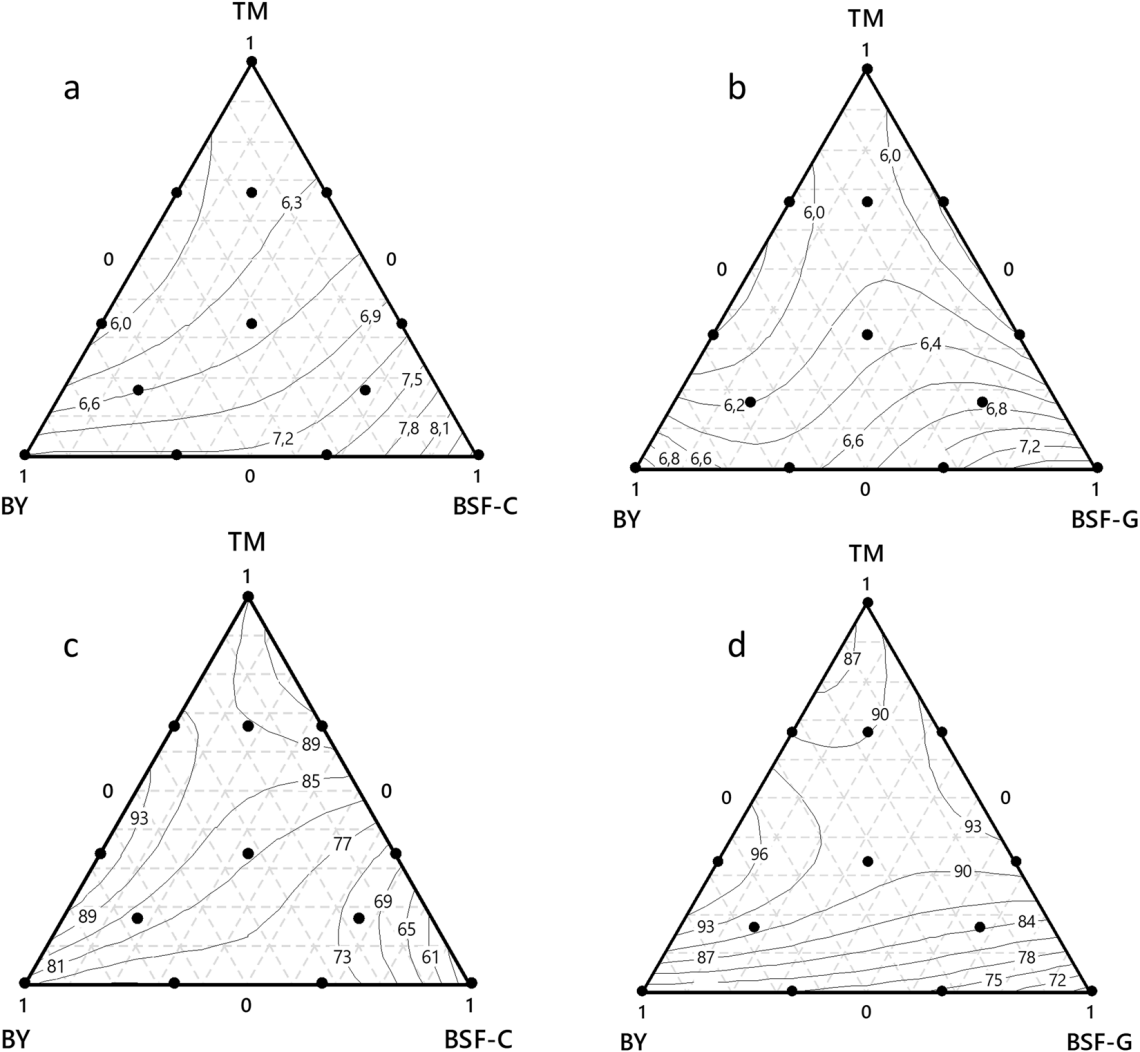
Effects of diet mixtures in each mixture design on *Aedes albopictus* larval development parameters. The top panels present the impact on larval development time from L1 to pupae in the mixtures TM, BY, BSF-C (**a**) and TM, BY, BSF-G (**b**). The bottom panels present the impact on larval survival rate from L1 to adults in the mixtures TM, BY, BSF-C (**c**) and TM, BY, BSF-G (**d**). In these plots, the response surface is viewed as a two-dimensional plane where all blends that have the same response are connected to produce contour lines of constant responses. TM: Tuna meal; BY: Brewer’s yeast; BSF-C: Black soldier fly corresponding to insect meal C; BSF-G: Black soldier fly corresponding to insect meal G.

All TM + BSF and TM + BY mixtures resulted in larval development times ranging between 5.58 and 7.2 days. The two-component blends BSF + BY induced the longest larval development times (>7.2 days) (Fig. 6a,b).

#### *Aedes albopictus* larval survival rate from L1 to adults

In the design with TM, BY, BSF-C (Fig. 6c), the two-component blends, 2/3 TM + 1/3 BY and 1/2TM + 1/2 BY resulted in the highest larval survival rate from L1 to adults (>91%), followed by 1/3 TM + 2/3 BY (91%), 2/3TM + 1/3BSF-C (87%) and 1/2TM + 1/2BSF-C (between 83–87%). In the design with TM, BY, BSF-G, the two-component blend 1/2TM + 1/2 BY induced the highest larval survival rate to adults (95%) followed by 2/3TM + 1/3BY, 1/3TM + 2/3BY, 2/3TM + 1/3BSF-G and 1/2TM + 1/2BSF-G (between 92 and 95%) and by 1/3 TM + 2/3BSF-G (89%) (Fig. 6d).

Overall, whatever the species and design, the centroid blends had the highest larval survival rate among the three-component blends.

### Effects of four diet mixtures on *Aedes albopictus* and *Aedes aegypti* larval and adult life history traits

Based on the results of the experiments 2 and 3, four mixtures were selected and assessed in regard to *Ae. albopictus* and *Ae. aegypti* larval and adult life history traits: Mix 1: 50% TM + 35% BSF + 15%BY; Mix 2: 1/3TM + 2/3BSF; Mix 3: 1/2TM + 1/2BSF; Mix 4: 2/3TM + 1/3BSF. The reference IAEA diet used as the control and was coded as Mix 0.

**Figure 7:**
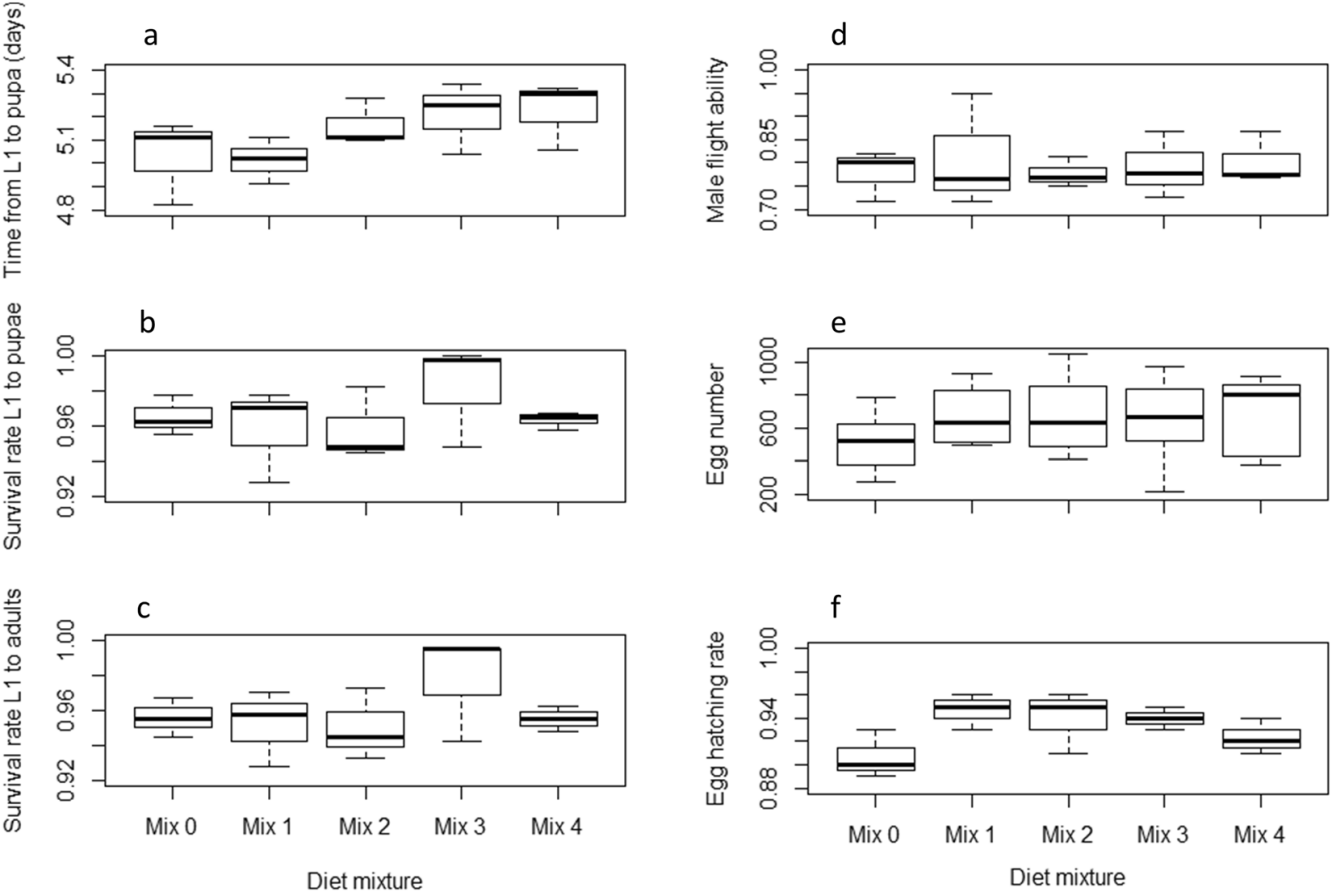
Effect of four diet mixtures on *Aedes aegypti* development time to pupation (**a**), survival rate to pupae (**b**) and to adults (**c**), flight ability (**d**), egg production (**e**) and egg hatch (**f**).

**Figure 8:**
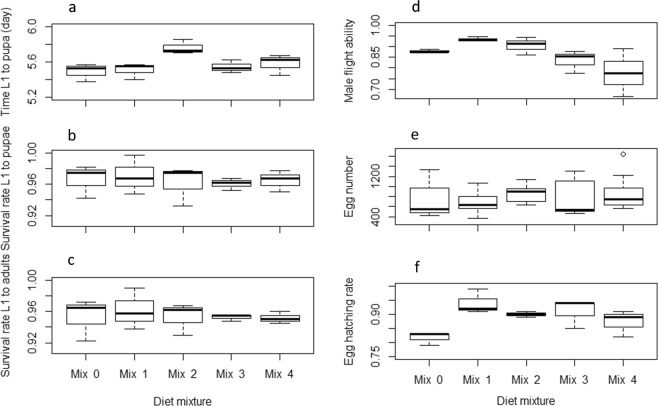
Effect of four diet mixtures on *Aedes albopictus* development time to pupation (**a**), survival rate to pupae (**b**) and to adults (**c**), flight ability (**d**), egg production (**e**) and egg hatch (**f**).

**Figure 9:**
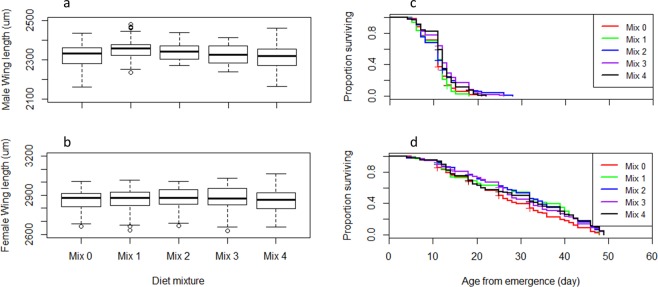
Effect of four diet mixtures on *Aedes albopictus* male (**a**) and female (**b**) wing length and male (**d**) and female (**e**) survival.

**Figure 10:**
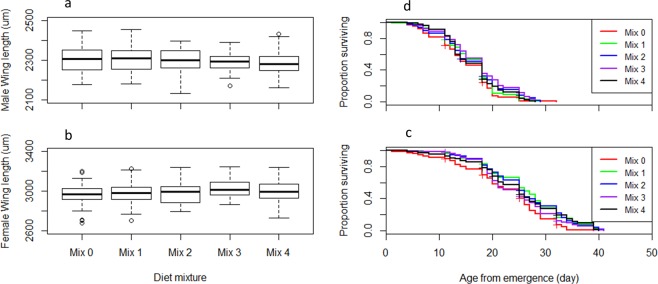
Effect of four diet mixtures on *Aedes aegypti* male (**a**) and female (**b**) wing length and male (**d**) and female (**e**) survival.

The effects of the tested diet mixtures on *Ae. albopictus* and *Ae. aegypti* larval and adult life history traits are summarized in Figs 7–10 and Tables S14–S19. Considering the larval development time, Mix 1 was similar to the control (*P* = 0.07) and the three other mixtures resulted in longer development times in *Ae. aegypti* (*P* < 0.05, Table S14, Fig. 7a) while only Mix 2 resulted in longer times and the other mixtures were similar to the control in *Ae. albopictus* (Table S17, Fig. 8). Even though significant differences were observed, differences in larval development times between diet mixtures were less than 1 day. Considering the other parameters including larval survival rates from L1 to pupae and to adults, male flight ability, egg production, egg hatch rate, adult body size and longevity, the assessed diet mixtures were similar or even enhanced effects compared to the control in both *Aedes* species, except Mix 4 which decreased the flight ability of male *Ae. albopictus* (Tables S14–S19). Considering all parameters and both species, Mix 1 and Mix 3 are preferred as Mix 1 was similar as the control in regards to all parameters, and Mix 3 was better or similar to the control except for *Ae. aegypti* larval development time.

## Discussion

The present study has demonstrated the feasibility of black soldier fly, yellow mealworm and house fly for breeding the main Zika and dengue vectors *Ae. aegypti* and *Ae. albopictus*. Indeed, pure insect meal, as larvae or pupae, defatted or not, or extracted proteins from BSF, YM or HF allowed these mosquito species to complete their development from L1 to adult mosquito with high pupation and emergence rates, regardless of the tested diet concentration. Interestingly, when used individually as a diet component substituting the bovine liver powder in the reference IAEA diet, the IM-based diet led to similar or even better results regarding larval survival compared to the control. The mixture design experiments using TM, BY and BSF (non-defatted or defatted meal) allowed eliciting optimal diet mixtures for both *Ae. albopictus* and *Ae. aegypti* most of which comprised only two ingredients such as the two blend mixtures 2/3TM + 1/3BSF, ½TM + ½BSF, 1/3TM + 2/3BSF, ½TM + ½BY, 2/3TM + 1/3BY and 1/3TM + 2/3BY. The further assessment of four insect-based mixtures in both *Aedes* species’ adults life history traits confirmed the effectiveness of these diets. Indeed, overall, the four assessed diets led to similar or enhanced effects compared to the control, especially the mixtures ½TM + ½BSF-C and 50%TM + 35%BSF-C + 15%BY. Even though only two BSF meals or only four mixtures were selected for deeper evaluation in our study, the other mixtures, meals or species are not disqualified as diet candidates and could be targeted in future investigations.

Insects are known to be a part of the natural or artificial diet for humans and animals especially in Asia, Latin America and Africa^36^. Black soldier fly, yellow mealworm, and house fly were previously found to be significant ingredients in the diet of livestock such us poultry, pig and fish^28^. However, our findings highlighted for the first time, that these insects are effective as pure diet or as a diet component for laboratory rearing of *Ae. albopictus* and *Ae. aegypti*, revealing the possibility of using insects as food for insects. This shows that they provide the required nutrients for the development of these mosquito species. Mosquito nutritional requirements are known to include protein (or amino acids), carbohydrate, fatty acids, nucleic acids, vitamins and mineral elements as indispensable, optional or optimal nutrients. Several studies have been devoted to the specific requirements, but this field remains unclear. Indeed, according to Singh and Brown^37^, L-valine, L-leucine, L-isoleucine, L-phenylalanine, L-histidine, l-arginine, L-tryptophan, L-threonine, L-methionine, and L-lysine are essential for *Ae. aegypti* larval growth, L-cystine is essential for pupation, L-proline, l-hydroxyproline and l-serine are required for normal growth and development while glycine, L-tyrosine, and L-alanine are not required. However, Goldberg and De Meillon^38^ have previously reported that glycine is indispensable to the same species and DL-phenylalanine and L-tyrosine were optimal for larval growth. In addition, previous studies reported that mosquito larvae must ingest long-chain polyunsaturated fatty acids (PUFA) such as arachidonic acid (AA; 20:4, n-6), eicosapentaenoic acid (EPA; 20:5, n-3), docosahexaenoic acid (DHA; 22:6; n-3), because of their inability to elongate the alpha-linolenic acid (ALA; 18:3; n-3) and linoleic acid (LA; 18:2; n-6), unlike most insects^39^. But Hood-Nowotny *et al*.^40^ have suggested *de-novo* synthesis in the 18C plus group in *An. arabiensis* and docosahexaenoic acid (DHA) was not an essential fatty acid. However, even though mosquito nutritional requirements still need to be elucidated, a direct ingestion of some elements from the larval diet is beneficial in terms of their importance in the structural and physiological functions. For example, the PUFAs, AA, EPA, DH are major components of the cell membrane, interact with the immune system and in reproductive functions, and enhance survival and flight activities^39,41–43^. It is also known that vitamins, especially thiamine, nicotinic acid, pyridoxine, calcium pantothenate, riboflavin, folic acid, biotin and choline, are important for optimal larval growth^44,45^ and carbohydrates, even if not indispensable for larval development, can optimize their growth rate. A direct ingestion of such nutrients from the diet is therefore beneficial.

The chemical analysis of the tested insect meals showed variable proportions of protein, fat and soluble sugar, based on the dry mass (Tables 2 and S20). This may rely on the process mode or on the feed quality during the breeding of the related insect^46^. As all animal products, the insect meal would contain all amino acids required for mosquito’s development. The high proportion of proteins would provide enough amino acids. The analysis of the fatty acids profile revealed variable proportions in SAT, MUFA and PUFA between the insect meals, including within species, with dominance in SAT in all of them except in the protein powder of YM where the MUFA were more represented. The specific profiles of MUFA or PUFA of the different ingredients raise questions about the mosquito larvae nutritional requirements. Indeed, LA, ALA, AA, EPA and DHP presumably known to be important or essential were not detected in most of the insect meals. EPA was found only in the housefly meal (IM 10-HFprot) whereas AA was absent in all insect meals. Only the DGLA, an intermediate fat acid in the synthesis of AA, was present in most insect meals. Considering the results from the evaluation of pure insect meal diets, the lack of these PUFAs suggests different hypotheses: they are not essential for *Ae. albopictus* and *Ae. aegypti* to complete their immature stage or *de novo* synthesis occurs^40^ or they are required only at vitamin concentration levels^47–49^ as previous studies have reported their presence. This lack would justify, partly, the longer larval development durations in the pure insect meal diets. The effectiveness of mixtures including tuna TM, IM and/or BY may result from the complementarity principle of nutrients from each component. Indeed, if the TM was found to have a similar profile of PUFAs as insect meals, the BY would provide LA, EPA, DGLA, AA and DHA. Together, they would compensate the nutritional requirements that could be provided by the BLP, including proteins, PUFAs and vitamins. This is highlighted by the striking similarity observed in all parameters of the mosquito larval development between the BLP-replaced-diet and the control. Therefore, the assessed BSF, YM and HF meal can be considered as effective substitutes of the BLP in the reference IAEA diet (1/2 TM + 7/20 BLP + 3/20 BY).

**Table 2:** Percentage of crude protein (%Nx6.25), soluble sugars fatty acids based on the dry weight of each diet ingredients.

Diet ingredients	% crude protein	% soluble sugars	% Fat	% MUFA	% PUFA	% SAT
IM A	62.1	1.1	52.8	6.3	11.1	35.4
IM B	61.6	1.0	32.8	7.4	9.7	15.8
IM C	45.8	0.3	66.73	14.2	18.4	34.2
IM D	32.8	0.0	71.14	11.0	15.3	44.8
IM F	31.2	1.0	74.2	7.8	23.8	50.0
IM G	58.3	9.7	28.8	7.8	7.1	13.9
IM H	56.5	0.5	40.5	10.6	11.3	18.7
IM I	53.7	0.0	40.2	9.7	15.2	15.3
IM J	55.2	4.4	15.9	1.3	4.5	10.1
TM	50.0	1.6	5.01	2.2	0.6	2.2
BLP	76.9	0.5	83.37	37.1	9.7	36.5
BY	59.3	0.4	77.8	10.2	13.9	55.0

IM E was not analysed; IM: Insect meal; TM: Tuna meal; BLP: Bovine liver powder; BY: Brewer’s yeast; MUFA: Monounsaturated fatty acid; PUFA: Polyunsaturated fatty acid; SAT: Saturated fatty acid./. The reference IAEA diet, despite its effectiveness is subjected to concerns. Indeed, the BLP is by far the most expensive ingredient as it costs 78 times more than the TM, 6 times more than the BY and alone comprises over 92% of the global cost of the mixture (Table 3). In addition, its current and future availability are not warranted. In contrast, edible insect products as feed are anticipated to cost $ USD 0.45 - $ USD 0.66 per kilogram live weight based on 35% dry matter and the maximal price should be $ USD1.12 - $ USD 1.67 per kg to be competitive^28^. This price is much lower than that of BLP ($ USD 63/kg), brewer’s yeast ($ USD 10/kg) and TM ($ USD 0.8/kg) without considering the expected increases in the future. Besides their effectiveness, the insect-based diet would therefore be much cheaper than the reference IAEA diet (more than 90% of cost reduction), particularly the two-component blends comprising of BSF and TM such as 2/3TM + 1/3BSF, ½TM + ½BSF and 1/3TM + 2/3BSF. Thus, considering all parameters, many insect-based mixtures can be recommended for the breeding of *Ae. albopictus* and *Ae. aegypti* and the optimal ones would be those that include only two ingredients. Insect meal is not only effective and affordable but also sustainable and is therefore suitable for the development of optimal mass-release- based techniques. Further investigations should address the adult life history traits including competitiveness, fecundity, fertility and longevity of the mosquitoes reared using optimal insect-based diets, such as ½TM + 7/20BSF + 3/20 BY, 2/3TM + 1/3BSF, ½TM + ½BSF, 1/3TM + 2/3BSF, in mass rearing conditions and over several generations.

**Table 3:** Reference IAEA diet ingredients, suppliers, proportion in the mixture and relative costs.

Supplier	Ingredient	Price of 1 kg ($USD)	Cost Ratios (BLP/ingredient)	Proportion (w/w) in the IAEA diet (%)	Account in the IAEA diet cost (%)
MP Biomedicals, Solon, OH	Bovine liver powder	63	1.00	35	92.1
T.C. Union Agrotech	Tuna meal	0.8	78.75	50	1.6
MP Biomedicals	Breyer’s yeast	10	6.3	15	6.3

BLP: Bovine Liver Powder; USD: United Stated dollars; IAEA: International Atomic Energy Agency.

## Conclusion

Our study pointed out for the first time that BSF, YM and HF meals can be effective ingredients in *Aedes* larval diet. Particularly, the diet mixtures 1/2TM + 7/20BSF + 3/20BY, 2/3TM + 1/3BSF, ½TM + ½BSF, 1/3TM + 2/3BSF were found optimal and cost-effective with regards to the reference IAEA diet. Our findings highlighted that edible insects, besides contributing to food security and environment protection, can play a major role in vector-borne disease control. This further promotes the use of insect products in general and therefore contributes to the achievement of the One Health concept, which is a global strategy integrating multidisciplinary efforts for optimal health for people, animals and environment.

## Materials and Methods

### Mosquito strains and colony rearing conditions

The strains of *Ae. aegypti* from Juazeiro, Brazil, and *Ae. albopictus* from Rimini, Italy, were used in this study. These strains were routinely maintained in a climate-controlled room at 26 ± 1 °C, 70 ± 10% RH, and a photoperiod of 12:12 (L:D), including 1 h dusk and 1 h dawn. Larvae were reared in plastic trays (30 × 40 × 8 cm) containing 1 liter of deionized water and fed with the reference IAEA diet (4% solution of 1/2TM + 7/20BSF + 3/20BY). Adults were loaded into 30 × 30 × 30 cm cages (Bioquip, Rancho Dominguez, Ca.) with constant access to 10% sugar solution. Fresh pig blood meals were provided to females for egg production.

#### Diet ingredients

The ingredients used in this study included tuna meal, bovine liver powder brewer’s yeast and ten insect meal. The different insect meal (thereafter A to J) are presented in Table 1. They were dry ground masses from black soldier fly, yellow mealworm or house fly, and differed also by the insect development instar (larvae or pre-pupae), the type of mass (non-defatted, defatted dry mass or protein powder) and the supplier.

#### Bioassay

Experiments 1 to 3 were performed using polystyrene Petri dishes (diameter 9 cm). Thirty-two first instar larvae (L1 < 4 h) were transferred into Petri dishes filled with 32 ml of deionized water. Five replicates were done for all treatments. For experiments 1 to 4, larvae were reared in a climate controlled room under T = 30 ± 2 °C, 70 ± 10% RH, and a photoperiod of 12:12 (L:D)h. Pupae were collected once per day, and every 24 h from the first collection, and transferred for emergence to a climate controlled room at T = 26 ± 1 °C, 70 ± 10% RH with a photoperiod of 12:12 (L:D)h.

### Experiment 1. Evaluation of pure insect meal diet on *Aedes albopictus* larval development

All insect meal (A-J) (Table 1) were assessed individually to elicit whether they could allow the larval development to adulthood. Three diet concentrations, 1%, 2% and 3%, named 1, 2 and 3, were assessed. Larvae were fed daily with 640 µl of diet solution per Petri dish. Larval development time from L1 to pupa and the larval survival rates from L1 to pupae and to adults were recorded.

### Experiment 2. Evaluation of insect meal as a substitute of BLP in the reference IAEA diet on *Aedes albopictus* larval development

Each IM was assessed as a “BLP replacement” in the reference IAEA diet, *e.g*. 1/2TM + 7/20BSF + 3/20BY. Treatments were named Mix A to Mix J, following the IM integration, and Mix K for the control (reference IAEA diet). Larvae were fed daily with 640 µl of 4% diet solution per Petri dish for all treatments. Larval development time from L1 to pupa and larval survival rates from L1 to pupae and to adults were determined.

### Experiment 3. Determination of optimal *Aedes* larval diet mixtures using tuna meal, brewer’s yeast and insect meal

Diet mixtures comprising of optimal proportions of TM, BY and IM (BSF-C or BSF-G) were determined through three-component mixture designs TM, BY and BSF-C and TM, BY and BSF-G. The augmented simplex lattice degree 3 design was considered to create mixtures with different proportions of TM, BY, BSF-C or of TM, BY, BSF-C as following: {1; 0; 0}; {0; 1; 0}; {0; 0; 1}; {1/3; 1/3; 1/3}; {2/3; 1/3; 0}; {2/3; 0; 1/3}; {1/3; 2/3; 0}; {0; 2/3; 1/3}; {1/3; 0; 2/3}; {0; 1/3; 2/3}; {2/3; 1/6; 1/6}; {1/6; 2/3; 1/6}; {1/6; 1/6; 2/3}. Larvae were fed daily with 640 µl of diet solution per Petri dish in all treatments. Only larval development time from L1 to pupa and larval survival rate from L1 to adults were recorded. The tests were performed on both *Ae. aegypti* and *Ae. albopictus* but at different times.

### Experiment 4. Assessment of four diet mixtures on *Aedes* larval and adult life history traits

The four diet mixtures were selected according to the results of the above experiments and assessed for both *Ae. aegypti* and *Ae. albopictus* larval and adult life history traits. These mixtures were encoded as Mix 1 to Mix 4 and the control was named as Mix 0.

Four hundred L1 were transferred into plastic trays (40 by 29 by 8 cm) containing 1 l of deionized water. Three replicates were performed for each diet mixtures. The 4% diet solution was provided daily, from day 0 (D0) to day 5 (D5), as following: 10 ml on D0, 10 ml in the morning + 20 ml in the evening on D1, 0 ml on D2 and 0 ml D3, 40 ml in the morning + 30 ml in the evening on D4 and 20 ml on D5). Larval development time from L1 to pupa and survival rates from L1 to pupa and to adult was recorded. Fifty males and fifty females, less than 24 h of age, from same batch of emergence were placed together in a plastic cage (30 × 30 × 30 cm), separately by replicate. Three batches of egg production were performed. Fresh pig blood meals were provided to females on two consecutive days, starting on the fourth day of age for the first batch and a week later for the next batch. Oviposition cups were placed in the cages on the second consecutive day after the second blood meal and removed on the following day and eggs counted. Egg hatch rate was checked for each treatment. At the same time, the longevity was measured by daily recording and removal of dead adults from the cages until all adults had died. A sugar solution (10%) was provided *ad libitum* to mosquitoes during the longevity and fecundity assessment. Adult body size was evaluated using the left-wing length. Fifteen males and 15 females were randomly selected from each replicate for wing dissections and measurements. Male flight ability was assessed, using the quality control device and protocol developed at the IPLC^50^.

### Statistical analysis

For experiments 1, 2 and 4, data were analyzed using R Software version 3.2.5. We used binomial generalized linear mixed models fit by maximum likelihood (Laplace Approximation) with the larval survival rates to pupae and to adults from the initial number of L1 as response variables, diet mixture as fix effect and the replicate as random effect. We used Gaussian linear mixed-effects model with the larval development time from L1 to pupa assigned as response variable, the diet mixture as fix effect and the replicate as random effect. The survival of adult mosquitoes reared on the different diets mixtures was analyzed using Kaplan-Meier survival curves. Survival curves were compared using the Cox proportional hazard model with the diet mixture as explanatory variable and survival rate as response variable. Data for experiment 3 were analyzed using Minitab 18 (Minitab^®^ Statistical Software, State College, PA).

## Supplementary information

Supplementary information

## Data Availability

All raw data are available as a Supplementary File.
