# Supplementary material for: Insects to feed insects - feeding Aedes mosquitoes with flies for laboratory rearing
Source: Sci Rep. 2019 Aug 6;9:11403. doi: 10.1038/s41598-019-47817-x (PMC6684809; doi:10.1038/s41598-019-47817-x)
Supplement: Supplementary file 1 — Supplementary information [file 41598_2019_47817_MOESM1_ESM.doc]

**Insects to feed insects - feeding *Aedes* mosquito with flies for laboratory rearing**

Nanwintoum Séverin Bimbilé Somda, Hamidou Maïga, Wadaka Mamai, Hanano Yamada, Adel Ali, Anna Konczal, Olivier Gnankiné, Abdoulaye Diabaté, Antoine Sanon, Kounbobr Roch Dabiré, Jérémie R.L. Gilles, Jérémy Bouyer

**Supplementary information**

**List of abbreviations**

IAEA: International Atomic Energy Agency

IM: Insect Meal

TM: Tuna Meal

BLP: Bovine liver powder

BY: Brewer’s Yeast

BSF: Black Soldier Fly

YM: Yellow Mealworm

HF: House Fly

BSF - C: Black Solider Fly, corresponding to insect meal C

BSF - G: Black Solider Fly, corresponding to insect meal G

Coef: Coefficient

Exp: Exponential

SE: Standard Error

DF: Degrees of Freedom

Ctrl: Control

Table S1. Effect of different concentrations of pure insect meal diet on *Aedes albopictus* larval survival rate from L1 to pupae (150 observations, 5 repeats).

|  | Estimate | SE | z value | Pr(>|z|) |
| --- | --- | --- | --- | --- |
| (Intercept) | 3.43399 | 0.45438 | 7.558 | 4.11e-14*** |
| IM A | -1.36853 | 0.51871 | -2.638 | 0.008331** |
| IM B | -1.42967 | 0.51593 | -2.771 | 0.005588** |
| IM C | -1.64950 | 0.50719 | -3.252 | 0.001145** |
| IM D | -1.92609 | 0.49858 | -3.863 | 0.000112*** |
| IM E | -1.23676 | 0.52527 | -2.355 | 0.018546* |
| IM F | -1.79424 | 0.50238 | -3.571 | 0.000355*** |
| IM G | -0.72594 | 0.55958 | -1.297 | 0.194531 |
| IM I | -0.34946 | 0.59654 | -0.586 | 0.558002 |
| IM J | -0.82794 | 0.55144 | -1.501 | 0.133248 |
| Concentration 1 | -0.72594 | 0.55958 | -1.297 | 0.194534 |
| Concentration 3 | -0.61393 | 0.56939 | -1.078 | 0.280928 |
| IM A: Concentration 1 | 0.79011 | 0.66449 | 1.189 | 0.234419 |
| IM B: Concentration 1 | 1.14710 | 0.67571 | 1.698 | 0.089579. |
| IM C: Concentration 1 | -0.02553 | 0.62944 | -0.041 | 0.967641 |
| IM D: Concentration 1 | 0.52796 | 0.62658 | 0.843 | 0.399449 |
| IM E: Concentration 1 | -0.08499 | 0.64934 | -0.131 | 0.895861 |
| IM F: Concentration 1 | 0.82079 | 0.63881 | 1.285 | 0.198833 |
| IM G: Concentration 1 | 1.10241 | 0.75445 | 1.461 | 0.143956 |
| IM I: Concentration 1 | -0.29314 | 0.72466 | -0.405 | 0.685833 |
| IM J: Concentration 1 | 1.20441 | 0.74843 | 1.609 | 0.107561 |
| IM A: Concentration 3 | 0.05638 | 0.65492 | 0.086 | 0.931399 |
| IM B: Concentration 3 | -0.11742 | 0.64846 | -0.181 | 0.856304 |
| IM C: Concentration 3 | 1.09813 | 0.66973 | 1.640 | 0.101077 |
| IM D: Concentration 3 | 0.65663 | 0.64000 | 1.026 | 0.304904 |
| IM E: Concentration 3 | -0.07539 | 0.66012 | -0.114 | 0.909073 |
| IM F: Concentration 3 | 0.61393 | 0.64502 | 0.952 | 0.341196 |
| IM G: Concentration 3 | -0.35952 | 0.69274 | -0.519 | 0.603775 |
| IM I: Concentration 3 | 0.96339 | 0.82465 | 1.168 | 0.242711 |
| IM J: Concentration 3 | 0.95232 | 0.74391 | 1.280 | 0.200490 |

IM H and Concentration 2 which apparently presented higher larval survival were considered as reference levels for regression.

Abbreviation: IM: Insect meal; SE: standard error.

Table S2. Effect of different concentrations of pure insect meal diet on *Aedes albopictus* larval survival rate from L1 to adults (150 observations, 5 repeats).

|  | Estimate | SE | z value | Pr(>|z|) |
| --- | --- | --- | --- | --- |
| (Intercept) | 3.24519 | 0.41611 | 7.799 | 6.25e-15 |
| IM A | -1.51059 | 0.47135 | -3.205 | 0.001351 |
| IM B | -1.55879 | 0.46964 | -3.319 | 0.000903 |
| IM C | -1.51059 | 0.47135 | -3.205 | 0.001351 |
| IM D | -1.81937 | 0.46169 | -3.941 | 8.13e-05 |
| IM E | -1.17974 | 0.48554 | -2.430 | 0.015109 |
| IM F | -1.69460 | 0.46523 | -3.642 | 0.000270 |
| IM G | -0.73289 | 0.51307 | -1.428 | 0.153167 |
| IM I | -0.42514 | 0.53934 | -0.788 | 0.430543 |
| IM J | -0.81971 | 0.50683 | -1.617 | 0.105807 |
| Concentration 1 | -0.81971 | 0.50683 | -1.617 | 0.105805 |
| Concentration 3 | -0.73289 | 0.51307 | -1.428 | 0.153165 |
| IM A: Concentration 1 | 1.03102 | 0.60252 | 1.711 | 0.087050 |
| IM B: Concentration 1 | 1.47786 | 0.61851 | 2.389 | 0.016877 |
| IM C: Concentration 1 | -0.03765 | 0.57967 | -0.065 | 0.948212 |
| IM D: Concentration 1 | 0.70381 | 0.57813 | 1.217 | 0.223455 |
| IM E: Concentration 1 | 0.02722 | 0.59669 | 0.046 | 0.963614 |
| IM F: Concentration 1 | 0.86362 | 0.58713 | 1.471 | 0.141309 |
| IM G: Concentration 1 | 1.39193 | 0.70453 | 1.976 | 0.048189 |
| IM I: Concentration 1 | -0.11039 | 0.65530 | -0.168 | 0.866218 |
| IM J: Concentration 1 | 1.47875 | 0.69999 | 2.113 | 0.034641 |
| IM A: Concentration 3 | 0.19955 | 0.58942 | 0.339 | 0.734943 |
| IM B: Concentration 3 | 0.07950 | 0.58560 | 0.136 | 0.892008 |
| IM C: Concentration 3 | 1.26697 | 0.62114 | 2.040 | 0.041377 |
| IM D: Concentration 3 | 0.81496 | 0.58768 | 1.387 | 0.165517 |
| IM E: Concentration 3 | 0.17533 | 0.60659 | 0.289 | 0.772545 |
| IM F: Concentration 3 | 0.31452 | 0.58347 | 0.539 | 0.589851 |
| IM G: Concentration 3 | -0.18491 | 0.63078 | -0.293 | 0.769417 |
| IM I: Concentration 3 | 0.18152 | 0.67419 | 0.269 | 0.787749 |
| IM J: Concentration 3 | 1.25184 | 0.69177 | 1.810 | 0.070353 |

IM H and Concentration 2 were considered as reference levels for regression since they presented higher larval survival rate.

Abbreviation: IM: Insect meal; SE: standard error.

Table S3. Effect of different concentrations of pure insect meal diet on *Aedes albopictus* larval development time (4242 observations, 5 repeats).

|  | Value | SE | DF | t-value | p-value |
| --- | --- | --- | --- | --- | --- |
| (Intercept) | 6.162601 | 0.09706503 | 4208 | 63.48941 | <0.001 |
| IM A | 1.663685 | 0.13275179 | 4208 | 12.53229 | <0.001 |
| IM B | 1.575218 | 0.13174719 | 4208 | 11.95637 | <0.001 |
| IM C | 0.565722 | 0.13274445 | 4208 | 4.26174 | <0.001 |
| IM D | 0.265839 | 0.13407476 | 4208 | 1.98277 | 0.0475 |
| IM E | -0.049884 | 0.13149440 | 4208 | -0.37936 | 0.7044 |
| IM F | -0.056068 | 0.13125348 | 4208 | -0.42717 | 0.6693 |
| IM G | 0.212236 | 0.12965167 | 4208 | 1.63697 | 0.1017 |
| IM I | -0.008693 | 0.12879561 | 4208 | -0.06750 | 0.9462 |
| IM J | -0.455957 | 0.12987646 | 4208 | -3.51070 | <0.001 |
| Concentration 1 | 0.750300 | 0.12943504 | 4208 | 5.79673 | <0.001 |
| Concentration 3 | -0.357749 | 0.12987883 | 4208 | -2.75448 | 0.0059 |
| IM A: Concentration 1 | 0.290128 | 0.18727929 | 4208 | 1.54917 | 0.1214 |
| IM B: Concentration 1 | 0.203615 | 0.18639028 | 4208 | 1.09241 | 0.2747 |
| IM C: Concentration 1 | 0.362112 | 0.19112236 | 4208 | 1.89466 | 0.0582 |
| IM D: Concentration 1 | -0.121655 | 0.19118821 | 4208 | -0.63631 | 0.5246 |
| IM E: Concentration 1 | 0.644007 | 0.18918234 | 4208 | 3.40416 | <0.001 |
| IM F: Concentration 1 | 0.650191 | 0.18901757 | 4208 | 3.43985 | <0.001 |
| IM G: Concentration 1 | 1.017872 | 0.18320199 | 4208 | 5.55601 | <0.001 |
| IM I: Concentration 1 | -0.207304 | 0.18518424 | 4208 | -1.11945 | 0.2630 |
| IM J: Concentration 1 | 0.742718 | 0.18410832 | 4208 | 4.03414 | <0.001 |
| IM A: Concentration 3 | 0.456626 | 0.18649865 | 4208 | 2.44841 | 0.0144 |
| IM B: Concentration 3 | 0.686046 | 0.18828604 | 4208 | 3.64364 | <0.001 |
| IM C: Concentration 3 | 0.217567 | 0.18693236 | 4208 | 1.16388 | 0.2445 |
| IM D: Concentration 3 | -0.302509 | 0.19050438 | 4208 | -1.58794 | 0.1124 |
| IM E: Concentration 3 | -0.283802 | 0.18908375 | 4208 | -1.50093 | 0.1334 |
| IM F: Concentration 3 | -0.253158 | 0.18851972 | 4208 | -1.34287 | 0.1794 |
| IM G: Concentration 3 | 0.086432 | 0.18663149 | 4208 | 0.46312 | 0.6433 |
| IM I: Concentration 3 | 0.384715 | 0.18291458 | 4208 | 2.10325 | 0.0355 |
| IM J: Concentration 3 | 0.210955 | 0.18474409 | 4208 | 1.14188 | 0.2536 |

IM H and Concentration 2 which apparently presented higher larval survival were considered as

reference levels for regression.

Abbreviation: IM: Insect meal; SE: standard error.

Table S4. Effect of insect meal as a substitution of BLP in the reference IAEA diet on *Aedes albopictus* larval development time to pupation in relation to the reference IAEA diet (1686 observations, 5 repeats)

|  | Value | SE | DF | t-value | p-value |
| --- | --- | --- | --- | --- | --- |
| (Intercept) | 5.241635 | 0.04542860 | 1671 | 115.38181 | <0.001*** |
| Mix A | 0.159327 | 0.05854959 | 1671 | 2.72123 | 0.0066 |
| Mix B | 0.229234 | 0.05755465 | 1671 | 3.98289 | <0.001*** |
| Mix C | -0.020794 | 0.05563744 | 1671 | -0.37374 | 0.7086 |
| Mix D | 0.162558 | 0.05746334 | 1671 | 2.82891 | 0.0047** |
| Mix E | 0.217831 | 0.05802918 | 1671 | 3.75381 | <0.001*** |
| Mix F | 0.033036 | 0.05746253 | 1671 | 0.57492 | 0.5654 |
| Mix G | 0.014292 | 0.05813113 | 1671 | 0.24586 | 0.8058 |
| Mix H | -0.079324 | 0.05832858 | 1671 | -1.35995 | 0.1740 |
| Mix I | 0.308466 | 0.05812664 | 1671 | 5.30680 | <0.001*** |
| Mix J | -0.176490 | 0.05833049 | 1671 | -3.02570 | 0.0025** |

Reference level for regression: Mix K.Ctrl (Reference IAEA diet).

Abbreviation: SE: standard error, DF: degree of freedom.

Significance codes: 0 ‘***’ 0.001 ‘**’ 0.01 ‘*’ 0.05 ‘.’ 0.1 ‘ ’ 1.

Table S5: Effect of insect meal as a substitution of BLP in the reference IAEA diet on *Aedes albopictus* larval survival rate in relation to the reference IAEA diet (55 observations, 5 repeats).

|  |  | Estimate | SE | z value | Pr(>|z|) |
| --- | --- | --- | --- | --- | --- |
| Survival rate L1 to pupae | (Intercept) | 2.9444 | 0.3627 | 8.117 | 4.77e-16 *** |
|  | Mix A | -0.5999 | 0.4581 | -1.310 | 0.1904 |
|  | Mix B | 0.4895 | 0.5814 | 0.842 | 0.3998 |
|  | Mix C | 2.1245 | 1.0667 | 1.992 | 0.0464 * |
|  | Mix D | 0.7191 | 0.6229 | 1.154 | 0.2483 |
|  | Mix E | -0.2364 | 0.4881 | -0.484 | 0.6282 |
|  | Mix F | 0.4895 | 0.5814 | 0.842 | 0.3998 |
|  | Mix G | -0.4321 | 0.4708 | -0.918 | 0.3587 |
|  | Mix H | -0.5190 | 0.4640 | -1.118 | 0.2634 |
|  | Mix I | -0.4321 | 0.4708 | -0.918 | 0.3587 |
|  | Mix J | -0.5190 | 0.4640 | -1.118 | 0.2634 |
| Survival rate L1 to adults | (Intercept) | 2.345e+00 | 2.798e-01 | 8.380 | < 2e-16 *** |
|  | Mix A | -1.473e-01 | 3.843e-01 | -0.383 | 0.70149 |
|  | Mix B | 9.006e-01 | 5.014e-01 | 1.796 | 0.07247 |
|  | Mix C | 2.025e+00 | 7.646e-01 | 2.648 | 0.00809 ** |
|  | Mix D | 9.006e-01 | 5.014e-01 | 1.796 | 0.07247 |
|  | Mix E | 3.635e-01 | 4.301e-01 | 0.845 | 0.39797 |
|  | Mix F | 1.089e+00 | 5.336e-01 | 2.042 | 0.04118 * |
|  | Mix G | -1.473e-01 | 3.843e-01 | -0.383 | 0.70149 |
|  | Mix H | -1.473e-01 | 3.843e-01 | -0.383 | 0.70149 |
|  | Mix I | 1.313e-14 | 3.957e-01 | 0.000 | 1.00000 |
|  | Mix J | 1.341e-14 | 3.957e-01 | 0.000 | 1.00000 |

Reference level for regression: Mix K.Ctrl (Reference IAEA diet).

Abbreviation: SE: standard error.

Significance codes : 0 ‘***’ 0.001 ‘**’ 0.01 ‘*’ 0.05.

Table S6. Regression for mixtures: *Aedes aegypti* larval survival rate versus TM, BY, BSF-C.

| Estimated Regression Coefficients | | | | | | |
| --- | --- | --- | --- | --- | --- | --- |
| Term | Coef | SE Coef | t-value | p-value | vif |  |
| TM | 82.76 | 3.42 | * | * | 2.34 |  |
| BY | 72.82 | 3.42 | * | * | 2.34 |  |
| BSF-C | 84.62 | 3.42 | * | * | 2.34 |  |
| TM*BY | 72.3 | 16.4 | 4.42 | <0.001 | 2.69 |  |
| TM*BSF-C | 56.4 | 16.4 | 3.45 | 0.001 | 2.69 |  |
| BY*BSF-C | 13.8 | 16.4 | 0.84 | 0.404 | 2.69 |  |
| TM*BY*BSF-C | -54 | 107 | -0.5 | 0.618 | 2.03 |  |
| | S | R-sq | R-sq(adj) | PRESS | R-sq(pred) | | --- | --- | --- | --- | --- | | 8.24073 | 45.51% | 39.88% | 5047.19 | 30.18% | | | | | | | |
| Analysis of Variance | | | | | | |
| Source | DF | Seq SS | Adj SS | Adj MS | F-Value | P-Value |
| Regression | 6 | 3289.91 | 3289.91 | 548.32 | 8.07 | <0.001 |
| Linear | 2 | 1217.22 | 506.08 | 253.04 | 3.73 | 0.03 |
| Quadratic | 3 | 2055.57 | 1750.87 | 583.62 | 8.59 | <0.001 |
| TM*BY | 1 | 1194.98 | 1324.17 | 1324.17 | 19.5 | <0.001 |
| TM*BSF-C | 1 | 827.64 | 807.89 | 807.89 | 11.9 | 0.001 |
| BY*BSF-C | 1 | 32.96 | 47.96 | 47.96 | 0.71 | 0.404 |
| Special Cubic | 1 | 17.12 | 17.12 | 17.12 | 0.25 | 0.618 |
| TM*BY*BSF-C | 1 | 17.12 | 17.12 | 17.12 | 0.25 | 0.618 |
| Residual Error | 58 | 3938.75 | 3938.75 | 67.91 |  |  |
| Lack-of-Fit | 6 | 520.79 | 520.79 | 86.8 | 1.32 | 0.265 |
| Pure Error | 52 | 3417.97 | 3417.97 | 65.73 |  |  |
| Total | 64 | 7228.67 |  |  |  |  |

TM: Tuna meal; BLP: Bovine liver powder; BY: Brewer’s yeast; BSF-C: Black soldier fly, corresponding to insect meal C.

Table S7. Regression for mixtures: *Aedes aegypti* larval survival rate versus TM, BY, BSF-G.

| Estimated Regression Coefficients | | | | | | |  |
| --- | --- | --- | --- | --- | --- | --- | --- |
| Term | Coef | | SE Coef | T-Value | P-Value | VIF |  |
| TM | 82.78 | | 2.75 | * | * | 2.34 |  |
| BY | 76.72 | | 2.75 | * | * | 2.34 |  |
| BSF-G | 88.58 | | 2.75 | * | * | 2.34 |  |
| TM*BY | 69.4 | | 13.1 | 5.28 | <0.001 | 2.69 |  |
| TM*BSF-G | 37.3 | | 13.1 | 2.84 | 0.006 | 2.69 |  |
| BY*BSF-G | 32.4 | | 13.1 | 2.47 | 0.017 | 2.69 |  |
| TM*BY*BSF-G | -22.5 | | 86.1 | -0.26 | 0.795 | 2.03 |  |
| | S | R-sq | R-sq(adj) | PRESS | R-sq(pred) | | --- | --- | --- | --- | --- | | 0.372487 | 61.37% | 57.37% | 10.4592 | 49.79% | | | | | | | | |
| Analysis of Variance | | | | | | | |
| Source | | DF | Seq SS | Adj SS | Adj MS | f-value | p-value |
| Regression | | 6 | 2080.07 | 2080.07 | 346.68 | 7.91 | <0.001 |
| Linear | | 2 | 323.77 | 442.54 | 221.27 | 5.05 | 0.009 |
| Quadratic | | 3 | 1753.33 | 1343.2 | 447.73 | 10.22 | <0.001 |
| TM*BY | | 1 | 1144.65 | 1220.75 | 1220.75 | 27.87 | <0.001 |
| TM*BSF-G | | 1 | 316.53 | 353.01 | 353.01 | 8.06 | 0.006 |
| BY*BSF-G | | 1 | 292.15 | 266.44 | 266.44 | 6.08 | 0.017 |
| Special Cubic | | 1 | 2.98 | 2.98 | 2.98 | 0.07 | 0.795 |
| TM*BY*BSF-G | | 1 | 2.98 | 2.98 | 2.98 | 0.07 | 0.795 |
| Residual Error | | 58 | 2540.42 | 2540.42 | 43.8 |  |  |
| Lack-of-Fit | | 6 | 317.76 | 317.76 | 52.96 | 1.24 | 0.302 |
| Pure Error | | 52 | 2222.66 | 2222.66 | 42.74 |  |  |
| Total | | 64 | 4620.49 |  |  |  |  |

TM: Tuna meal; BLP: Bovine liver powder; BY: Brewer’s yeast; BSF-G: Black soldier fly, corresponding to insect meal G.

Table S8. Regression for mixtures: *Aedes aegypti* larval development time from L1 to pupation versus TM, BY, BSF-C.

| Estimated Regression Coefficients | | | | | | |
| --- | --- | --- | --- | --- | --- | --- |
| Term | Coef | SE Coef | t-value | p-value | vif |  |
| TM | 6.511 | 0.155 | * | * | 2.34 |  |
| BY | 6.144 | 0.155 | * | * | 2.34 |  |
| BSF-C | 6.779 | 0.155 | * | * | 2.34 |  |
| TM*BY | -3.698 | 0.74 | -5 | <0.001 | 2.69 |  |
| TM*BSF-C | -4.226 | 0.74 | -5.71 | <0.001 | 2.69 |  |
| BY*BSF-C | 0.571 | 0.74 | 0.77 | 0.443 | 2.69 |  |
| TM*BY*BSF-C | 0.1 | 4.84 | 0.02 | 0.983 | 2.03 |  |
| | S | R-sq | R-sq(adj) | PRESS | R-sq(pred) | | --- | --- | --- | --- | --- | | 0.372487 | 61.37% | 57.37% | 10.4592 | 49.79% | | | | | | | |
| Analysis of Variance | | | | | | |
| Source | DF | Seq SS | Adj SS | Adj MS | F-Value | P-Value |
| Regression | 6 | 12.7827 | 12.7827 | 2.13045 | 15.35 | <0.001 |
| Linear | 2 | 3.6298 | 1.2801 | 0.64007 | 4.61 | 0.014 |
| Quadratic | 3 | 9.1529 | 7.7708 | 2.59028 | 18.67 | <0.001 |
| TM*BY | 1 | 3.3724 | 3.4673 | 3.46734 | 24.99 | <0.001 |
| TM*BSF-C | 1 | 5.6794 | 4.5281 | 4.52814 | 32.64 | <0.001 |
| BY*BSF-C | 1 | 0.1011 | 0.0826 | 0.08263 | 0.6 | 0.443 |
| Special Cubic | 1 | 0.0001 | 0.0001 | 0.00006 | <0.001 | 0.983 |
| TM*BY*BSF-C | 1 | 0.0001 | 0.0001 | 0.00006 | <0.001 | 0.983 |
| Residual Error | 58 | 8.0473 | 8.0473 | 0.13875 |  |  |
| Lack-of-Fit | 6 | 1.2538 | 1.2538 | 0.20896 | 1.6 | 0.166 |
| Pure Error | 52 | 6.7935 | 6.7935 | 0.13064 |  |  |
| Total | 64 | 20.83 |  |  |  |  |

TM: Tuna meal; BLP: Bovine liver powder; BY: Brewer’s yeast; BSF-C: Black soldier fly, corresponding to insect meal C.

Table S9. Regression for mixtures: *Aedes aegypti* larval development time from L1 to pupation versus TM, BY, BSF-G.

| Estimated Regression Coefficients | | | | | |  |
| --- | --- | --- | --- | --- | --- | --- |
| Term | Coef | SE Coef | T-Value | P-Value | vif |  |
| TM | 6.555 | 0.11 | * | * | 2.34 |  |
| BY | 6.113 | 0.11 | * | * | 2.34 |  |
| BSF-G | 6.508 | 0.11 | * | * | 2.34 |  |
| TM*BY | -3.538 | 0.525 | -6.74 | <0.001 | 2.69 |  |
| TM*BSF-G | -4.058 | 0.525 | -7.73 | <0.001 | 2.69 |  |
| BY*BSF-G | -1.071 | 0.525 | -2.04 | 0.046 | 2.69 |  |
| TM*BY*BSF-G | -1.16 | 3.44 | -0.34 | 0.737 | 2.03 |  |
| | S | R-sq | R-sq(adj) | PRESS | R-sq(pred) | | --- | --- | --- | --- | --- | | 0.264442 | 68.38% | 65.11% | 5.06940 | 60.48% | | | | | | | |
| Analysis of Variance | | | | | | |
| Source | DF | Seq SS | Adj SS | Adj MS | F-Value | P-Value |
| Regression | 6 | 8.7706 | 8.77062 | 1.46177 | 20.9 | <0.001 |
| Linear | 2 | 0.6097 | 0.7406 | 0.3703 | 5.3 | 0.008 |
| Quadratic | 3 | 8.1529 | 5.89516 | 1.96505 | 28.1 | <0.001 |
| TM*BY | 1 | 2.8548 | 3.17445 | 3.17445 | 45.39 | <0.001 |
| TM*BSF-G | 1 | 4.9017 | 4.17589 | 4.17589 | 59.72 | <0.001 |
| BY*BSF-G | 1 | 0.3964 | 0.2908 | 0.2908 | 4.16 | 0.046 |
| Special Cubic | 1 | 0.008 | 0.00798 | 0.00798 | 0.11 | 0.737 |
| TM*BY*BSF-G | 1 | 0.008 | 0.00798 | 0.00798 | 0.11 | 0.737 |
| Residual Error | 58 | 4.0559 | 4.05591 | 0.06993 |  |  |
| Lack-of-Fit | 6 | 0.7244 | 0.72445 | 0.12074 | 1.88 | 0.101 |
| Pure Error | 52 | 3.3315 | 3.33146 | 0.06407 |  |  |
| Total | 64 | 12.8265 |  |  |  |  |

TM: Tuna meal; BLP: Bovine liver powder; BY: Brewer’s yeast; BSF-G: Black soldier fly, corresponding to insect meal G.

Table S10. Regression for mixtures: *Aedes albopictus* larval survival rate versus TM, BY, BSF-C.

| Estimated Regression Coefficients | | | | | | |
| --- | --- | --- | --- | --- | --- | --- |
| Term | Coef | SE Coef | T-Value | P-Value | VIF |  |
| TM | 88.52 | 5.37 | * | * | 2.34 |  |
| BY | 78.14 | 5.37 | * | * | 2.34 |  |
| BSF-C | 54.03 | 5.37 | * | * | 2.34 |  |
| TM*BY | 45.3 | 25.7 | 1.76 | 0.083 | 2.69 |  |
| TM*BSF-C | 45.5 | 25.7 | 1.77 | 0.082 | 2.69 |  |
| BY*BSF-C | 37.3 | 25.7 | 1.45 | 0.152 | 2.69 |  |
| TM*BY*BSF-C | -170 | 168 | -1.01 | 0.316 | 2.03 |  |
| | S | R-sq | R-sq(adj) | PRESS | R-sq(pred) | | --- | --- | --- | --- | --- | | 12.9373 | 42.80% | 36.88% | 12696.3 | 25.19% | | | | | | | |
| Analysis of Variance | | | | | | |
| Source | DF | Seq SS | Adj SS | Adj MS | F-Value | P-Value |
| Regression | 6 | 7263.5 | 7263.5 | 1210.6 | 7.23 | <0.001 |
| Linear | 2 | 6323.8 | 3940 | 1970 | 11.77 | <0.001 |
| Quadratic | 3 | 768.6 | 917 | 305.7 | 1.83 | 0.152 |
| TM*BY | 1 | 242.7 | 519.9 | 519.9 | 3.11 | 0.083 |
| TM*BSF-C | 1 | 308.6 | 525.5 | 525.5 | 3.14 | 0.082 |
| BY*BSF-C | 1 | 217.3 | 352.7 | 352.7 | 2.11 | 0.152 |
| Special Cubic | 1 | 171.1 | 171.1 | 171.1 | 1.02 | 0.316 |
| TM*BY*BSF-C | 1 | 171.1 | 171.1 | 171.1 | 1.02 | 0.316 |
| Residual Error | 58 | 9707.7 | 9707.7 | 167.4 |  |  |
| Lack-of-Fit | 6 | 754.5 | 754.5 | 125.8 | 0.73 | 0.627 |
| Pure Error | 52 | 8953.1 | 8953.1 | 172.2 |  |  |
| Total | 64 | 16971.2 |  |  |  |  |

TM: Tuna meal; BLP: Bovine liver powder; BY: Brewer’s yeast; BSF-C: Black soldier fly, corresponding to insect meal C.

Table S11. Regression for mixtures: *Aedes albopictus* larval survival rate versus TM, BY, BSF-G.

| Estimated Regression Coefficients | | | | | | |
| --- | --- | --- | --- | --- | --- | --- |
| Term | Coef | SE Coef | T-Value | P-Value | VIF |  |
| TM | 85.05 | 4.27 | * | * | 2.34 |  |
| BY | 80.47 | 4.27 | * | * | 2.34 |  |
| BSF-G | 68.24 | 4.27 | * | * | 2.34 |  |
| TM*BY | 50.7 | 20.4 | 2.48 | 0.016 | 2.69 |  |
| TM*BSF-G | 70.1 | 20.4 | 3.43 | 0.001 | 2.69 |  |
| BY*BSF-G | 11.4 | 20.4 | 0.56 | 0.579 | 2.69 |  |
| TM*BY*BSF-G | -35 | 134 | -0.26 | 0.794 | 2.03 |  |
| | S | R-sq | R-sq(adj) | PRESS | R-sq(pred) | | --- | --- | --- | --- | --- | | 10.2911 | 41.12% | 35.02% | 7704.58 | 26.14% | | | | | | | |
| Analysis of Variance | | | | | | |
| Source | DF | Seq SS | Adj SS | Adj MS | F-Value | P-Value |
| Regression | 6 | 4289.2 | 4289.17 | 714.86 | 6.75 | <0.001 |
| Linear | 2 | 2382.9 | 950.44 | 475.22 | 4.49 | 0.015 |
| Quadratic | 3 | 1899 | 1581.04 | 527.01 | 4.98 | 0.004 |
| TM*BY | 1 | 498.8 | 652.41 | 652.41 | 6.16 | 0.016 |
| TM*BSF-G | 1 | 1374.3 | 1245.68 | 1245.68 | 11.76 | 0.001 |
| BY*BSF-G | 1 | 25.9 | 32.98 | 32.98 | 0.31 | 0.579 |
| Special Cubic | 1 | 7.3 | 7.28 | 7.28 | 0.07 | 0.794 |
| TM*BY*BSF-G | 1 | 7.3 | 7.28 | 7.28 | 0.07 | 0.794 |
| Residual Error | 58 | 6142.6 | 6142.62 | 105.91 |  |  |
| Lack-of-Fit | 6 | 1170 | 1169.97 | 194.99 | 2.04 | 0.077 |
| Pure Error | 52 | 4972.7 | 4972.66 | 95.63 |  |  |
| Total | 64 | 10431.8 |  |  |  |  |

TM: Tuna meal; BLP: Bovine liver powder; BY: Brewer’s yeast; BSF-G: Black soldier fly, corresponding to insect meal G.

Table S12. Regression for mixtures: *Ae. albopictus* larval development time from L1 to pupation versus TM, BY, BSF-C.

| Estimated Regression Coefficients | | | | | | |
| --- | --- | --- | --- | --- | --- | --- |
| Term | Coef | SE Coef | T-Value | P-Value | VIF |  |
| TM | 6.235 | 0.275 | * | * | 2.34 |  |
| BY | 7.241 | 0.275 | * | * | 2.34 |  |
| BSF-C | 8.712 | 0.275 | * | * | 2.34 |  |
| TM*BY | -3.82 | 1.32 | -2.9 | 0.005 | 2.69 |  |
| TM*BSF-C | -3.35 | 1.32 | -2.54 | 0.014 | 2.69 |  |
| BY*BSF-C | -2.89 | 1.32 | -2.2 | 0.032 | 2.69 |  |
| TM*BY*BSF-C | 6.14 | 8.63 | 0.71 | 0.48 | 2.03 |  |
| | S | R-sq | R-sq(adj) | PRESS | R-sq(pred) | | --- | --- | --- | --- | --- | | 0.663640 | 60.84% | 56.79% | 33.9720 | 47.92% | | | | | | | |
| Analysis of Variance | | | | | | |
| Source | DF | Seq SS | Adj SS | Adj MS | F-Value | P-Value |
| Regression | 6 | 39.6817 | 39.6817 | 6.6136 | 15.02 | <0.001 |
| Linear | 2 | 32.8675 | 19.5322 | 9.7661 | 22.17 | <0.001 |
| Quadratic | 3 | 6.5917 | 5.7131 | 1.9044 | 4.32 | 0.008 |
| TM*BY | 1 | 2.4987 | 3.6978 | 3.6978 | 8.4 | 0.005 |
| TM*BSF-C | 1 | 2.1763 | 2.8475 | 2.8475 | 6.47 | 0.014 |
| BY*BSF-C | 1 | 1.9168 | 2.1222 | 2.1222 | 4.82 | 0.032 |
| Special Cubic | 1 | 0.2225 | 0.2225 | 0.2225 | 0.51 | 0.48 |
| TM*BY*BSF-C | 1 | 0.2225 | 0.2225 | 0.2225 | 0.51 | 0.48 |
| Residual Error | 58 | 25.5442 | 25.5442 | 0.4404 |  |  |
| Lack-of-Fit | 6 | 1.9899 | 1.9899 | 0.3317 | 0.73 | 0.626 |
| Pure Error | 52 | 23.5543 | 23.5543 | 0.453 |  |  |
| Total | 64 | 65.2259 |  |  |  |  |

TM: Tuna meal; BLP: Bovine liver powder; BY: Brewer’s yeast; BSF-C: Black soldier fly, corresponding to insect meal C.

Table S13. Regression for mixtures: *Aedes albopictus* larval development time from L1 to pupation versus TM, BY, BSF-G.

| Estimated Regression Coefficients | | | | | |  |
| --- | --- | --- | --- | --- | --- | --- |
| Term | Coef | SE Coef | t-value | p-value | VIF |  |
| TM | 6.118 | 0.171 | * | * | 2.71 |  |
| BY | 7.235 | 0.171 | * | * | 2.71 |  |
| BSF-G | 7.556 | 0.171 | * | * | 2.71 |  |
| TM*BY | -3.73 | 0.763 | -4.89 | <0.001 | 2.72 |  |
| TM*BSF-G | -4.245 | 0.763 | -5.57 | <0.001 | 2.72 |  |
| BY*BSF-G | -2.553 | 0.763 | -3.35 | 0.001 | 2.72 |  |
| TM*BY*BSF-G | 13.79 | 4.97 | 2.77 | 0.008 | 2.03 |  |
| TM*BY*(-) | 2.98 | 1.46 | 2.04 | 0.046 | 1.25 |  |
| TM*BSF-G*(-) | 1.48 | 1.46 | 1.01 | 0.315 | 1.25 |  |
| BY*BSF-G*(-) | -3.16 | 1.46 | -2.16 | 0.035 | 1.25 |  |
| | S | R-sq | R-sq(adj) | PRESS | R-sq(pred) | | --- | --- | --- | --- | --- | | 0.382254 | 71.68% | 67.05% | 11.1024 | 60.88% | | | | | | | |
| Analysis of Variance | | | | | | |
| Source | DF | Seq SS | Adj SS | Adj MS | F-Value | P-Value |
| Regression | 9 | 20.3445 | 20.3445 | 2.2605 | 15.47 | <0.001 |
| Linear | 2 | 11.6918 | 5.7093 | 2.8546 | 19.54 | <0.001 |
| Quadratic | 3 | 5.9053 | 6.6039 | 2.2013 | 15.07 | <0.001 |
| TM*BY | 1 | 1.5674 | 3.4966 | 3.4966 | 23.93 | <0.001 |
| TM*BSF-G | 1 | 3.6253 | 4.5286 | 4.5286 | 30.99 | <0.001 |
| BY*BSF-G | 1 | 0.7126 | 1.6381 | 1.6381 | 11.21 | 0.001 |
| Special Cubic | 1 | 1.124 | 1.124 | 1.124 | 7.69 | 0.008 |
| TM*BY*BSF-G | 1 | 1.124 | 1.124 | 1.124 | 7.69 | 0.008 |
| Full Cubic | 3 | 1.6234 | 1.6234 | 0.5411 | 3.7 | 0.017 |
| TM*BY*(-) | 1 | 0.8583 | 0.6081 | 0.6081 | 4.16 | 0.046 |
| TM*BSF-G*(-) | 1 | 0.0828 | 0.1505 | 0.1505 | 1.03 | 0.315 |
| BY*BSF-G*(-) | 1 | 0.6823 | 0.6823 | 0.6823 | 4.67 | 0.035 |
| Residual Error | 55 | 8.0365 | 8.0365 | 0.1461 |  |  |
| Lack-of-Fit | 3 | 0.8791 | 0.8791 | 0.293 | 2.13 | 0.108 |
| Pure Error | 52 | 7.1574 | 7.1574 | 0.1376 |  |  |
| Total | 64 | 28.381 |  |  |  |  |

TM: Tuna meal; BLP: Bovine liver powder; BY: Brewer’s yeast; BSF-G: Black soldier fly, corresponding to Insect meal G.

Table S14. Effect of four insect-based diets on *Aedes aegypti* larval development time, egg production and adult body size in relation to the reference IAEA diet.

|  |  | Value | SE | DF | t-value | p-value |
| --- | --- | --- | --- | --- | --- | --- |
| Time from L1 to pupa | (Intercept) | 5.030000 | 0.08107610 | 8 | 62.04048 | <0.001 |
|  | Mix 1 | -0.016667 | 0.05530722 | 8 | -0.30135 | 0.7708 |
|  | Mix 2 | 0.133333 | 0.05530722 | 8 | 2.41078 | 0.0425 * |
|  | Mix 3 | 0.180000 | 0.05530722 | 8 | 3.25455 | 0.0116 * |
|  | Mix 4 | 0.196667 | 0.05530722 | 8 | 3.55589 | 0.0074 ** |
| Egg production | (Intercept) | 582.5556 | 96.34494 | 32 | 6.046561 | <0.001 |
|  | Mix 1 | 105.4444 | 99.77107 | 32 | 1.056864 | 0.2985 |
|  | Mix 2 | 93.2222 | 99.77107 | 32 | 0.934361 | 0.3571 |
|  | Mix 3 | 57.3333 | 99.77107 | 32 | 0.574649 | 0.5695 |
|  | Mix 4 | 106.5556 | 99.77107 | 32 | 1.068001 | 0.2935 |
| Male wing length | (Intercept) | 2305.8182 | 9.418112 | 218 | 244.82806 | <0.001 |
|  | Mix 1 | -1.2281 | 13.319222 | 218 | -0.09220 | 0.9266 |
|  | Mix 2 | -12.1593 | 13.319222 | 218 | -0.91292 | 0.3623 |
|  | Mix 3 | -19.5940 | 13.319222 | 218 | -1.47111 | 0.1427 |
|  | Mix 4 | -20.6551 | 13.319222 | 218 | -1.55077 | 0.1224 |
| Female wing length | (Intercept) | 2974.1338 | 19.75897 | 218 | 150.52067 | <0.001 |
|  | Mix 1 | -15.8018 | 27.94341 | 218 | -0.56549 | 0.5723 |
|  | Mix 2 | -0.5176 | 27.94341 | 218 | -0.01852 | 0.9852 |
|  | Mix 3 | 81.0922 | 27.94341 | 218 | 2.90202 | 0.0041 ** |
|  | Mix 4 | 19.9838 | 27.94341 | 218 | 0.71515 | 0.475 |

Reference level for regression: Mix 0 (Reference IAEA diet).

Abbreviation: SE: standard error; DF: degree of freedom.

Significance codes: 0 ‘***’ 0.001 ‘**’ 0.01 ‘*’ 0.05.

Table S15. Effect of four insect-based diets on *Aedes aegypti* larval survival rates, male flight ability and egg hatch rate in relation to the reference IAEA diet.

|  |  | Estimate | SE | z value | Pr(>|z|) |
| --- | --- | --- | --- | --- | --- |
| Survival rate L1 to pupae | (Intercept) | 3.32619 | 0.17783 | 18.705 | <2e-16 *** |
|  | Mix 1 | -0.18141 | 0.21326 | -0.851 | 0.3949 |
|  | Mix 2 | -0.18141 | 0.21326 | -0.851 | 0.3949 |
|  | Mix 3 | 0.66407 | 0.26618 | 2.495 | 0.0126 * |
|  | Mix 4 | -0.04828 | 0.21953 | -0.220 | 0.8259 |
| Survival rate L1 to adults | (Intercept) | 3.07589 | 0.14389 | 21.376 | < 2e-16 *** |
|  | Mix 1 | -0.09454 | 0.19456 | -0.486 | 0.62703 |
|  | Mix 2 | -0.13019 | 0.19309 | -0.674 | 0.50013 |
|  | Mix 3 | 0.69694 | 0.24005 | 2.903 | 0.00369 ** |
|  | Mix 4 | -0.01957 | 0.19781 | -0.099 | 0.92121 |
| Male flight ability | (Intercept) | 1.28698 | 0.24124 | 5.335 | 9.56e-08 *** |
|  | Mix 1 | 0.19294 | 0.26419 | 0.730 | 0.465 |
|  | Mix 2 | -0.01916 | 0.25803 | -0.074 | 0.941 |
|  | Mix 3 | 0.07010 | 0.26021 | 0.269 | 0.788 |
|  | Mix 4 | 0.14061 | 0.26293 | 0.535 | 0.593 |

Reference level for regression:: Mix 0 (Reference IAEA diet).

Abbreviation: SE: standard error.

Significance codes : 0 ‘***’ 0.001 ‘**’ 0.01 ‘*’ 0.05.

Table S16. Effect of four insect-based diets on *Aedes albopictus* larval development time, egg production and adult body size in relation to the reference IAEA diet.

|  |  | Value | SE | DF | t-value | p-value |
| --- | --- | --- | --- | --- | --- | --- |
| Time from L1 to pupa | (Intercept) | 5.490000 | 0.05501515 | 8 | 99.79069 | <0.001 |
|  | Mix 1 | 0.016667 | 0.07154641 | 8 | 0.23295 | 0.8217 |
|  | Mix 2 | 0.273333 | 0.07154641 | 8 | 3.82036 | 0.0051** |
|  | Mix 3 | 0.053333 | 0.07154641 | 8 | 0.74544 | 0.4773 |
|  | Mix 4 | 0.090000 | 0.07154641 | 8 | 1.25792 | 0.2439 |
| Egg production | (Intercept) | 754.0000 | 127.9597 | 32 | 5.892481 | 0.0000 |
|  | Mix 1 | -68.1111 | 115.6358 | 32 | -0.589014 | 0.5600 |
|  | Mix 2 | 116.0000 | 115.6358 | 32 | 1.003150 | 0.3233 |
|  | Mix 3 | 39.4444 | 115.6358 | 32 | 0.341109 | 0.7353 |
|  | Mix 4 | 133.6667 | 115.6358 | 32 | 1.155928 | 0.2563 |
| Male wing length | (Intercept) | 2325.8018 | 9.099315 | 218 | 255.60187 | <0.001 |
|  | Mix 1 | 34.1540 | 11.976712 | 218 | 2.85170 | 0.0048** |
|  | Mix 2 | 17.6293 | 11.976712 | 218 | 1.47197 | 0.1425 |
|  | Mix 3 | 2.2156 | 11.976712 | 218 | 0.18499 | 0.8534 |
|  | Mix 4 | -8.2991 | 11.976712 | 218 | -0.69294 | 0.4891 |
| Female wing length | (Intercept) | 2861.4249 | 61.95180 | 218 | 46.18792 | <0.001 |
|  | Mix 1 | 5.0429 | 87.61307 | 218 | 0.05756 | 0.9542 |
|  | Mix 2 | 144.7044 | 87.61307 | 218 | 1.65163 | 0.1000 |
|  | Mix 3 | 9.4024 | 87.61307 | 218 | 0.10732 | 0.9146 |
|  | Mix 4 | 1.7920 | 87.61307 | 218 | 0.02045 | 0.9837 |

Reference level for regression: Mix 0 (Reference IAEA diet).

Abbreviation: SE: standard error, DF: degree of freedom.

Significance codes : 0 ‘***’ 0.001 ‘**’ 0.01 ‘*’ 0.05 ‘.’ 0.1 ‘ ’ 1.

Table S17. Effect of four insect-based diets on *Aedes albopictus* larval survival rates, male flight ability and egg hatch rate in relation to the reference IAEA diet.

|  |  | Estimate | SE | z value | Pr(>|z|) |
| --- | --- | --- | --- | --- | --- |
| Larval survival rate L1 to pupae | (Intercept) | 3.36730 | 0.16082 | 20.939 | <2e-16 *** |
|  | Mix 1 | 0.13783 | 0.23514 | 0.586 | 0.558 |
|  | Mix 2 | -0.14495 | 0.22015 | -0.658 | 0.510 |
|  | Mix 3 | -0.16732 | 0.21910 | -0.764 | 0.445 |
|  | Mix 4 | -0.05052 | 0.22480 | -0.225 | 0.822 |
| Larval survival rate L1 to adults | (Intercept) | 3.017e+00 | 1.369e-01 | 22.044 | <2e-16 *** |
|  | Mix 1 | 2.054e-01 | 2.033e-01 | 1.010 | 0.312 |
|  | Mix 2 | -8.424e-15 | 1.936e-01 | 0.000 | 1.000 |
|  | Mix 3 | -1.857e-02 | 1.927e-01 | -0.096 | 0.923 |
|  | Mix 4 | -3.684e-02 | 1.920e-01 | -0.192 | 0.848 |
| Male flight ability | (Intercept) | 1.9865 | 0.2476 | 8.022 | 1.04e-15 *** |
|  | Mix 1 | 0.6841 | 0.3927 | 1.742 | 0.0815 |
|  | Mix 2 | 0.2839 | 0.3549 | 0.800 | 0.4238 |
|  | Mix 3 | -0.3524 | 0.3183 | -1.107 | 0.2682 |
|  | Mix 4 | -0.7325 | 0.3015 | -2.429 | 0.0151* |
| Egg hatching rate | (Intercept) | 2.1607 | 0.1897 | 11.392 | <2e-16 *** |
|  | Mix 1 | 0.3759 | 0.2916 | 1.289 | 0.197 |
|  | Mix 2 | 0.0365 | 0.2702 | 0.135 | 0.893 |
|  | Mix 3 | 0.4260 | 0.2953 | 1.443 | 0.149 |
|  | Mix 4 | 0.1129 | 0.2745 | 0.411 | 0.681 |

Reference level for regression: Mix 0 (Reference IAEA diet).

Abbreviation: SE: standard error.

Significance codes : 0 ‘***’ 0.001 ‘**’ 0.01 ‘*’ 0.05.

Table S18. Effect of four insect-based diets on *Aedes aegypti* male and female risk of mortality in relation to the reference IAEA diet.

|  | Diet | Coef | Exp(coef) | SE(coef) | Z | Pr(>|Z|) |
| --- | --- | --- | --- | --- | --- | --- |
| Male | Mix 1 | -0.2322 | 0.7928 | 0.1336 | -1.738 | 0.082284 |
|  | Mix 2 | -0.3599 | 0.6977 | 0.1347 | -2.671 | 0.007556 ** |
|  | Mix 3 | -0.4846 | 0.6159 | 0.1344 | -3.605 | 0.000312 *** |
|  | Mix 4 | -0.2213 | 0.8015 | 0.1333 | -1.660 | 0.096860 |
| Female | Mix 1 | -0.5254 | 0.5913 | 0.1294 | -4.060 | 4.92e-05 *** |
|  | Mix 2 | -0.4986 | 0.6074 | 0.1293 | -3.857 | 0.000115 *** |
|  | Mix 3 | -0.6244 | 0.5356 | 0.1309 | -4.771 | 1.84e-06 *** |
|  | Mix 4 | -0.4438 | 0.6416 | 0.1299 | -3.416 | 0.000635 *** |

Reference level for regression: Mix 0 (Reference IAEA diet).

Abbreviation: Coef: Coefficient; Exp: Exponential; SE : standard error.

Significance codes : 0 ‘***’ 0.001 ‘**’ 0.01 ‘*’ 0.05 ‘.’ 0.1 ‘ ’ 1

Table S19. Effect of four candidate diets on *Aedes albopictus* male and female survival in relation to the reference IAEA diet.

|  | Diet | Coef | Exp(coef) | SE(Coef) | Z | Pr(>|Z|) |
| --- | --- | --- | --- | --- | --- | --- |
| Male | Mix 1 | 0.05903 | 1.06081 | 0.14142 | 0.417 | 0.67637 |
|  | Mix 2 | -0.39572 | 0.67319 | 0.14201 | -2.787 | 0.00533 ** |
|  | Mix 3 | -0.56304 | 0.56947 | 0.14034 | -4.012 | 6.02e-05 *** |
|  | Mix 4 | -0.41044 | 0.66336 | 0.13964 | -2.939 | 0.00329 ** |
| Female | Mix 1 | -0.3525 | 0.7030 | 0.1263 | -2.791 | 0.00525 ** |
|  | Mix 2 | -0.3448 | 0.7084 | 0.1258 | -2.740 | 0.00614 ** |
|  | Mix 3 | -0.2817 | 0.7545 | 0.1262 | -2.232 | 0.02561 * |
|  | Mix 4 | -0.3306 | 0.7185 | 0.1269 | -2.605 | 0.00919 ** |

Reference level for regression: Mix 0 (Reference IAEA diet).

Abbreviation: Coef: Coefficient; Exp: Exponential; SE: standard error.

Significance codes : 0 ‘***’ 0.001 ‘**’ 0.01 ‘*’ 0.05 ‘.’ 0.1 ‘ ’ 1.

Table S20. Profile and proportion of fatty acid, based on a total of 32 studied fatty acids (weight: weight), in each diet ingredient.

| Fatty acid | Type | | Name | IM A | | IM B | | IM C | | IM D | | IM F | | IM G | | IM H | IM I | | IM J | TM | BLP | | BY | |  | |
| --- | --- | --- | --- | --- | --- | --- | --- | --- | --- | --- | --- | --- | --- | --- | --- | --- | --- | --- | --- | --- | --- | --- | --- | --- | --- | --- |
| Myristoleic acid | MUFA | | 14:1w5c | 0.14 | | 0.10 | | 0.27 | | 0.16 | | 0.15 | | 0.12 | | 0.00 | 0.21 | | 0.40 | 0.75 | 0.11 | | 0.05 | |  | |
| Pentadecanoic acid* | MUFA | | 15:1w5c | 0.00 | | 0.00 | | 0.00 | | 0.00 | | 0.00 | | 0.00 | | 0.00 | 0.00 | | 0.07 | 0.00 | 0.04 | | 0.00 | |  | |
| Palmitoleic acid | MUFA | | 16:1w7 | 5.62 | | 3.01 | | 2.13 | | 3.54 | | 3.63 | | 2.24 | | 1.37 | 0.45 | | 12.16 | 34.59 | 0.73 | | 0.34 | |  | |
| Heptadecenoic acid* | MUFA | | 17:1w7 | 0.00 | | 0.06 | | 0.07 | | 0.00 | | 0.09 | | 0.00 | | 0.33 | 0.15 | | 0.36 | 0.00 | 0.70 | | 0.69 | |  | |
| Oleic acid | MUFA | | 18:1w9c | 15.41 | | 8.79 | | 17.14 | | 16.49 | | 9.69 | | 17.25 | | 36.18 | 45.83 | | 0.00 | 8.69 | 5.74 | | 16.73 | |  | |
| Gadoleic acid | MUFA | | 20:1 w9c | 0.11 | | 0.15 | | 0.39 | | 0.00 | | 0.12 | | 0.26 | | 0.53 | 0.41 | | 0.38 | 0.00 | 0.70 | | 0.55 | |  | |
| Erucic acid | MUFA | | 22:1w9 | 0.00 | | 0.00 | | 0.00 | | 0.00 | | 0.00 | | 0.00 | | 0.00 | 0.00 | | 0.17 | 0.00 | 0.00 | | 0.42 | |  | |
| Nervonic acid | MUFA | | 24:1w9 | 0.00 | | 0.00 | | 0.00 | | 0.00 | | 0.00 | | 0.00 | | 0.00 | 0.00 | | 0.00 | 0.00 | 0.10 | | 0.00 | |  | |
| **Gamma-Linolenic acid (GLA)** | **PUFA** | | **18:3w3c** | **0.56** | | **0.22** | | **0.12** | | **0.00** | | **0.21** | | **0.00** | | **0.31** | **0.00** | | **0.00** | **0.00** | **0.13** | | **0.00** | |  | |
| **Linoleic acid c (LA)** | **PUFA** | | **18:2w6c** | **0.00** | | **0.00** | | **0.00** | | **10.02** | | **0.00** | | **0.00** | | **12.21** | **0.00** | | **0.00** | **0.00** | **0.00** | | **0.76** | |  | |
| **Linoleic acid t** | **PUFA** | | **18:2w6t** | **26.90** | | **15.70** | | **31.35** | | **0.29** | | **17.70** | | **7.49** | | **0.53** | **23.36** | | **39.50** | **11.48** | **9.39** | | **3.20** | |  | |
| **Eicosapentaenoic acid (EPA)** | **PUFA** | | **20:5w3** | **0.00** | | **0.00** | | **0.00** | | **0.00** | | **0.00** | | **0.00** | | **0.00** | **0.00** | | **0.08** | **0.00** | **0.00** | | **1.13** | |  | |
| **Arachidonic acid (AA)** | **PUFA** | | **20:4w6** | **0.00** | | **0.00** | | **0.00** | | **0.00** | | **0.00** | | **0.00** | | **0.00** | **0.00** | | **0.00** | **0.00** | **10.29** | | **1.24** | |  | |
| Eicosatrienoic acid | PUFA | | 20:3w3 | 0.14 | | 0.00 | | 0.00 | | 0.00 | | 0.00 | | 0.19 | | 1.18 | 0.13 | | 0.00 | 0.00 | 1.22 | | 0.26 | |  | |
| Eicosadienoic acid | PUFA | | 20:2w6 | 0.00 | | 0.00 | | 0.00 | | 0.00 | | 0.00 | | 0.00 | | 0.00 | 0.00 | | 0.10 | 0.00 | 2.84 | | 0.00 | |  | |
| **Dihomo-gamma-linolenic acid (DGLA)** | **PUFA** | | **20:3w6** | **0.07** | | **0.06** | | **0.05** | | **0.00** | | **0.05** | | **0.18** | | **0.60** | **0.18** | | **0.59** | **0.53** | **0.45** | | **1.02** | |  | |
| **Docosahexaenoic acid (DHA)** | **PUFA** | | **22:6w3** | **0.00** | | **0.00** | | **0.00** | | **0.00** | | **0.00** | | **0.00** | | **0.41** | **0.00** | | **0.00** | **0.00** | **0.29** | | **0.61** | |  | |
| **Docosadienoic acid** | **PUFA** | | **22:2w6** | **0.00** | | **0.00** | | **0.00** | | **0.00** | | **0.00** | | **0.00** | | **0.00** | **0.92** | | **0.02** | **0.00** | **0.00** | | **0.28** | |  | |
| Capric acid | SAT | | .10:0 | 1.84 | | 0.87 | | 2.34 | | 0.97 | | 0.85 | | 1.33 | | 0.41 | 0.01 | | 2.39 | 24.29 | 0.02 | | 0.00 | |  | |
| Undecylic acid | SAT | | .11:0 | 0.02 | | 0.02 | | 0.03 | | 0.02 | | 0.01 | | 0.03 | | 0.00 | 0.02 | | 0.03 | 0.05 | 0.01 | | 0.00 | |  | |
| Lauric acid | SAT | | .12:0 | 0.00 | | 41.56 | | 15.56 | | 39.83 | | 39.34 | | 39.91 | | 1.90 | 0.24 | | 0.00 | 15.13 | 0.05 | | 0.09 | |  | |
| Tridecylic acid | SAT | | .13:0 | 0.02 | | 0.01 | | 0.00 | | 0.00 | | 0.01 | | 0.01 | | 0.11 | 0.05 | | 0.08 | 0.00 | 0.08 | | 0.42 | |  | |
| Myristic acid | SAT | | .14:0 | 13.78 | | 7.13 | | 17.18 | | 6.39 | | 6.88 | | 6.59 | | 4.00 | 1.93 | | 13.01 | 3.79 | 1.62 | | 3.53 | |  | |
| Pentadecanoic acid* | SAT | | .15:0 | 0.10 | | 0.06 | | 0.13 | | 0.06 | | 0.06 | | 0.17 | | 0.32 | 0.14 | | 0.95 | 0.10 | 0.39 | | 1.93 | |  | |
| Palmitic acid | SAT | | .16:0 | 29.42 | | 18.44 | | 10.78 | | 18.08 | | 17.39 | | 19.81 | | 26.32 | 16.54 | | 0.00 | 0.00 | 33.94 | | 45.11 | |  | |
| Argaric acid | SAT | | .17:0 | 0.24 | | 0.13 | | 0.11 | | 0.16 | | 0.14 | | 0.30 | | 0.66 | 0.48 | | 0.81 | 0.00 | 1.60 | | 2.85 | |  | |
| Stearic acid | SAT | | .18:0 | 5.49 | | 3.61 | | 2.33 | | 3.77 | | 3.50 | | 3.94 | | 11.45 | 8.87 | | 28.24 | 0.00 | 28.65 | | 18.07 | |  | |
| Arachidic acid | SAT | | .20:0 | 0.14 | | 0.09 | | 0.00 | | 0.23 | | 0.14 | | 0.18 | | 0.74 | 0.10 | | 0.33 | 0.61 | 0.49 | | 0.33 | |  | |
| Heneicosanoic acid | SAT | | .21:0 | 0.00 | | 0.00 | | 0.00 | | 0.00 | | 0.00 | | 0.00 | | 0.00 | 0.00 | | 0.03 | 0.00 | 0.00 | | 0.00 | |  | |
| Behenic acid | SAT | | .22:0 | 0.01 | | 0.01 | | 0.00 | | 0.00 | | 0.05 | | 0.02 | | 0.42 | 0.00 | | 0.16 | 0.00 | 0.44 | | 0.37 | |  | |
| Tricosanoic acid | SAT | | .23:0 | 0.00 | | 0.00 | | 0.00 | | 0.00 | | 0.00 | | 0.00 | | 0.00 | 0.00 | | 0.00 | 0.00 | 0.00 | | 0.00 | |  | |
| Lignoceric acid | SAT | | .24:0 | 0.00 | | 0.00 | | 0.00 | | 0.00 | | 0.00 | | 0.00 | | 0.00 | 0.00 | | 0.14 | 0.00 | 0.00 | | 0.00 | |  | |
| Proportions of each type of saturation in the total amount of fatty acid | | | | | | | | | | | | | | | | | | | | | | | | | |  |
| MUFA |  |  | | | 21.28 | | 12.11 | | 20.01 | | 20.19 | | 13.67 | | 19.87 | 13.67 | | 47.05 | 13.54 | 44.03 | | 8.12 | | 18.78 | | |
| PUFA |  |  | | | 27.10 | | 15.76 | | 31.40 | | 10.30 | | 17.75 | | 7.85 | 17.75 | | 24.58 | 40.30 | 12.01 | | 24.47 | | 8.50 | | |
| SAT |  |  | | | 51.06 | | 71.91 | | 48.46 | | 69.50 | | 68.37 | | 72.28 | 68.37 | | 28.38 | 46.16 | 43.96 | | 67.28 | | 72.71 | | |

*NB: IM E was not analysed.*
